# Supplementary material for: Influence of transient pressure changes on speech intelligibility: Implications for next-generation train travel
Source: PLoS One. 2020 Apr 23;15(4):e0232024. doi: 10.1371/journal.pone.0232024 (PMC7179854; doi:10.1371/journal.pone.0232024)
Supplement: S1 File — (PDF) [file pone.0232024.s001.pdf]

<u>Supplement</u>

Influence of transient pressure changes on speech intelligibility: implications for next-generation train travel

Daniel Rooney, Martin Wittkowski, Susanne Bartels, Sarah Weidenfeld, Daniel Aeschbach

The following table provides the raw data recorded during the experiment. The variables are:

Observation: Index of the observation (arbitrary).

Subject: Identifies the different participants.

Trial: Identifies the test word played, i.e. each participant heard the same test word during trial 1, trial 2, etc. Test words were played in ascending order of this trial number.

Correct: Indicates whether the participant selected the correct test word on the tablet.

Pressure: Amplitude of the pressure change preceding the playback of the test word.

Observation	Subject	Trial	Correct	Pressure
1	1	1	1	0
2	1	2	0	0
3	1	3	1	0
4	1	4	1	0
5	1	5	0	0
6	1	6	0	0
7	1	7	1	0
8	1	8	0	0
9	1	9	1	0
10	1	10	1	0
11	1	11	0	0
12	1	12	1	0
13	1	13	0	0
14	1	14	0	0
15	1	15	1	0
16	1	16	1	0
17	1	17	1	0
18	1	18	1	0
19	1	19	0	0
20	1	20	0	0
21	1	21	0	0
22	1	22	1	0
23	1	23	0	0

Observation	Subject	Trial	Correct	Pressure
24	1	24	1	0
25	1	25	1	0
26	1	26	0	0
27	1	27	1	0
28	1	28	1	0
29	1	29	0	0
30	1	30	0	0
31	1	31	1	0
32	1	32	0	0
33	1	33	0	0
34	1	34	1	0
35	1	35	0	0
36	1	36	0	0
37	1	37	1	0
38	1	38	0	0
39	1	39	0	0
40	1	40	1	0
41	1	41	1	0
42	1	42	0	0
43	1	43	0	0
44	1	44	0	0
45	1	45	1	0
46	1	46	0	0
47	1	47	1	0
48	1	48	0	0
49	1	49	1	0
50	1	50	0	0
51	1	51	1	-25
52	1	52	0	25
53	1	53	0	-25
54	1	54	0	-25
55	1	55	0	25
56	1	56	0	-25
57	1	57	1	25
58	1	58	1	-25
59	1	59	1	25
60	1	60	0	25
61	1	61	1	-25
62	1	62	1	25
63	1	63	1	-25
64	1	64	0	-25
65	1	65	1	25
66	1	66	0	-25
67	1	67	1	25

Observation	Subject	Trial	Correct	Pressure
68	1	68	1	25
69	1	69	1	-25
70	1	70	1	-25
71	1	71	1	25
72	1	72	0	-25
73	1	73	0	25
74	1	74	0	-25
75	1	75	0	25
76	1	76	0	25
77	1	77	1	-25
78	1	78	0	25
79	1	79	0	-25
80	1	80	1	25
81	1	81	0	-25
82	1	82	0	25
83	1	83	1	-25
84	1	84	1	-25
85	1	85	1	25
86	1	86	1	25
87	1	87	0	-25
88	1	88	0	25
89	1	89	1	-25
90	1	90	0	-25
91	1	91	0	25
92	1	92	0	-25
93	1	93	1	25
94	1	94	1	-25
95	1	95	1	25
96	1	96	1	25
97	1	97	1	-25
98	1	98	1	25
99	1	99	1	-25
100	1	100	0	25
101	2	1	1	0
102	2	2	1	0
103	2	3	1	0
104	2	4	1	0
105	2	5	0	0
106	2	6	1	0
107	2	7	1	0
108	2	8	0	0
109	2	9	1	0
110	2	10	1	0
111	2	11	1	0

Observation	Subject	Trial	Correct	Pressure
112	2	12	1	0
113	2	13	1	0
114	2	14	0	0
115	2	15	1	0
116	2	16	1	0
117	2	17	0	0
118	2	18	1	0
119	2	19	0	0
120	2	20	0	0
121	2	21	1	0
122	2	22	1	0
123	2	23	0	0
124	2	24	1	0
125	2	25	1	0
126	2	26	1	0
127	2	27	0	0
128	2	28	1	0
129	2	29	0	0
130	2	30	0	0
131	2	31	1	0
132	2	32	0	0
133	2	33	0	0
134	2	34	1	0
135	2	35	0	0
136	2	36	0	0
137	2	37	1	0
138	2	38	0	0
139	2	39	1	0
140	2	40	1	0
141	2	41	1	0
142	2	42	1	0
143	2	43	1	0
144	2	44	1	0
145	2	45	1	0
146	2	46	0	0
147	2	47	1	0
148	2	48	1	0
149	2	49	1	0
150	2	50	0	0
151	2	51	1	-25
152	2	52	1	25
153	2	53	1	-25
154	2	54	1	-25
155	2	55	0	25

Observation	Subject	Trial	Correct	Pressure
156	2	56	0	-25
157	2	57	1	25
158	2	58	0	-25
159	2	59	0	25
160	2	60	0	25
161	2	61	1	-25
162	2	62	1	25
163	2	63	1	-25
164	2	64	0	-25
165	2	65	0	25
166	2	66	0	-25
167	2	67	1	25
168	2	68	0	25
169	2	69	1	-25
170	2	70	0	-25
171	2	71	1	25
172	2	72	1	-25
173	2	73	1	25
174	2	74	0	-25
175	2	75	0	25
176	2	76	0	25
177	2	77	1	-25
178	2	78	1	25
179	2	79	1	-25
180	2	80	1	25
181	2	81	0	-25
182	2	82	1	25
183	2	83	1	-25
184	2	84	1	-25
185	2	85	0	25
186	2	86	1	25
187	2	87	0	-25
188	2	88	0	25
189	2	89	1	-25
190	2	90	0	-25
191	2	91	0	25
192	2	92	0	-25
193	2	93	1	25
194	2	94	0	-25
195	2	95	0	25
196	2	96	1	25
197	2	97	1	-25
198	2	98	0	25
199	2	99	1	-25

Observation	Subject	Trial	Correct	Pressure
200	2	100	1	25
201	3	1	1	0
202	3	2	1	0
203	3	3	1	0
204	3	4	1	0
205	3	5	0	0
206	3	6	1	0
207	3	7	1	0
208	3	8	0	0
209	3	9	1	0
210	3	10	1	0
211	3	11	0	0
212	3	12	0	0
213	3	13	1	0
214	3	14	0	0
215	3	15	1	0
216	3	16	0	0
217	3	17	0	0
218	3	18	1	0
219	3	19	1	0
220	3	20	0	0
221	3	21	1	0
222	3	22	1	0
223	3	23	1	0
224	3	24	1	0
225	3	25	0	0
226	3	26	1	0
227	3	27	0	0
228	3	28	1	0
229	3	29	0	0
230	3	30	0	0
231	3	31	1	0
232	3	32	0	0
233	3	33	0	0
234	3	34	1	0
235	3	35	1	0
236	3	36	0	0
237	3	37	1	0
238	3	38	0	0
239	3	39	0	0
240	3	40	1	0
241	3	41	1	0
242	3	42	0	0
243	3	43	1	0

Observation	Subject	Trial	Correct	Pressure
244	3	44	1	0
245	3	45	1	0
246	3	46	1	0
247	3	47	1	0
248	3	48	0	0
249	3	49	0	0
250	3	50	1	0
251	3	51	0	-25
252	3	52	1	25
253	3	53	1	-25
254	3	54	1	-25
255	3	55	1	25
256	3	56	1	-25
257	3	57	1	25
258	3	58	1	-25
259	3	59	0	25
260	3	60	0	25
261	3	61	1	-25
262	3	62	1	25
263	3	63	1	-25
264	3	64	0	-25
265	3	65	1	25
266	3	66	0	-25
267	3	67	1	25
268	3	68	0	25
269	3	69	1	-25
270	3	70	0	-25
271	3	71	1	25
272	3	72	0	-25
273	3	73	1	25
274	3	74	0	-25
275	3	75	0	25
276	3	76	0	25
277	3	77	1	-25
278	3	78	1	25
279	3	79	0	-25
280	3	80	1	25
281	3	81	0	-25
282	3	82	1	25
283	3	83	1	-25
284	3	84	0	-25
285	3	85	0	25
286	3	86	1	25
287	3	87	0	-25

Observation	Subject	Trial	Correct	Pressure
288	3	88	1	25
289	3	89	1	-25
290	3	90	0	-25
291	3	91	1	25
292	3	92	0	-25
293	3	93	1	25
294	3	94	0	-25
295	3	95	1	25
296	3	96	1	25
297	3	97	0	-25
298	3	98	0	25
299	3	99	1	-25
300	3	100	1	25
301	4	1	1	0
302	4	2	0	0
303	4	3	1	0
304	4	4	1	0
305	4	5	0	0
306	4	6	1	0
307	4	7	1	0
308	4	8	0	0
309	4	9	1	0
310	4	10	1	0
311	4	11	1	0
312	4	12	0	0
313	4	13	1	0
314	4	14	0	0
315	4	15	1	0
316	4	16	1	0
317	4	17	0	0
318	4	18	1	0
319	4	19	1	0
320	4	20	0	0
321	4	21	0	0
322	4	22	1	0
323	4	23	1	0
324	4	24	1	0
325	4	25	0	0
326	4	26	0	0
327	4	27	0	0
328	4	28	1	0
329	4	29	0	0
330	4	30	0	0
331	4	31	1	0

Observation	Subject	Trial	Correct	Pressure
332	4	32	1	0
333	4	33	0	0
334	4	34	1	0
335	4	35	1	0
336	4	36	0	0
337	4	37	1	0
338	4	38	1	0
339	4	39	0	0
340	4	40	1	0
341	4	41	1	0
342	4	42	0	0
343	4	43	1	0
344	4	44	1	0
345	4	45	1	0
346	4	46	0	0
347	4	47	0	0
348	4	48	1	0
349	4	49	0	0
350	4	50	0	0
351	4	51	1	-25
352	4	52	0	25
353	4	53	1	-25
354	4	54	1	-25
355	4	55	0	25
356	4	56	0	-25
357	4	57	0	25
358	4	58	1	-25
359	4	59	1	25
360	4	60	0	25
361	4	61	1	-25
362	4	62	1	25
363	4	63	1	-25
364	4	64	1	-25
365	4	65	0	25
366	4	66	0	-25
367	4	67	1	25
368	4	68	0	25
369	4	69	1	-25
370	4	70	1	-25
371	4	71	1	25
372	4	72	0	-25
373	4	73	1	25
374	4	74	0	-25
375	4	75	1	25

Observation	Subject	Trial	Correct	Pressure
376	4	76	0	25
377	4	77	0	-25
378	4	78	1	25
379	4	79	0	-25
380	4	80	1	25
381	4	81	0	-25
382	4	82	1	25
383	4	83	1	-25
384	4	84	0	-25
385	4	85	1	25
386	4	86	1	25
387	4	87	0	-25
388	4	88	0	25
389	4	89	0	-25
390	4	90	1	-25
391	4	91	1	25
392	4	92	1	-25
393	4	93	1	25
394	4	94	0	-25
395	4	95	1	25
396	4	96	0	25
397	4	97	1	-25
398	4	98	1	25
399	4	99	1	-25
400	4	100	1	25
401	5	1	1	-25
402	5	2	0	25
403	5	3	1	-25
404	5	4	1	-25
405	5	5	1	25
406	5	6	1	-25
407	5	7	1	25
408	5	8	1	-25
409	5	9	1	25
410	5	10	1	25
411	5	11	1	-25
412	5	12	1	25
413	5	13	0	-25
414	5	14	0	-25
415	5	15	1	25
416	5	16	0	-25
417	5	17	0	25
418	5	18	1	25
419	5	19	1	-25

Observation	Subject	Trial	Correct	Pressure
420	5	20	1	-25
421	5	21	1	25
422	5	22	0	-25
423	5	23	1	25
424	5	24	1	-25
425	5	25	0	25
426	5	26	0	25
427	5	27	0	-25
428	5	28	1	25
429	5	29	0	-25
430	5	30	1	25
431	5	31	1	-25
432	5	32	0	25
433	5	33	0	-25
434	5	34	1	-25
435	5	35	1	25
436	5	36	0	25
437	5	37	1	-25
438	5	38	0	25
439	5	39	1	-25
440	5	40	1	-25
441	5	41	1	25
442	5	42	1	-25
443	5	43	1	25
444	5	44	0	-25
445	5	45	1	25
446	5	46	1	25
447	5	47	0	-25
448	5	48	1	25
449	5	49	1	-25
450	5	50	0	25
451	5	51	0	0
452	5	52	0	0
453	5	53	0	0
454	5	54	1	0
455	5	55	0	0
456	5	56	0	0
457	5	57	1	0
458	5	58	0	0
459	5	59	0	0
460	5	60	1	0
461	5	61	1	0
462	5	62	1	0
463	5	63	1	0

Observation	Subject	Trial	Correct	Pressure
464	5	64	1	0
465	5	65	1	0
466	5	66	1	0
467	5	67	1	0
468	5	68	0	0
469	5	69	1	0
470	5	70	1	0
471	5	71	1	0
472	5	72	1	0
473	5	73	1	0
474	5	74	0	0
475	5	75	1	0
476	5	76	0	0
477	5	77	1	0
478	5	78	0	0
479	5	79	1	0
480	5	80	1	0
481	5	81	0	0
482	5	82	1	0
483	5	83	1	0
484	5	84	1	0
485	5	85	0	0
486	5	86	1	0
487	5	87	0	0
488	5	88	0	0
489	5	89	1	0
490	5	90	0	0
491	5	91	1	0
492	5	92	0	0
493	5	93	1	0
494	5	94	0	0
495	5	95	1	0
496	5	96	1	0
497	5	97	1	0
498	5	98	1	0
499	5	99	1	0
500	5	100	0	0
501	6	1	1	-25
502	6	2	0	25
503	6	3	0	-25
504	6	4	1	-25
505	6	5	0	25
506	6	6	0	-25
507	6	7	1	25

Observation	Subject	Trial	Correct	Pressure
508	6	8	1	-25
509	6	9	1	25
510	6	10	0	25
511	6	11	1	-25
512	6	12	0	25
513	6	13	0	-25
514	6	14	0	-25
515	6	15	1	25
516	6	16	1	-25
517	6	17	0	25
518	6	18	0	25
519	6	19	0	-25
520	6	20	0	-25
521	6	21	1	25
522	6	22	1	-25
523	6	23	1	25
524	6	24	1	-25
525	6	25	0	25
526	6	26	0	25
527	6	27	1	-25
528	6	28	1	25
529	6	29	0	-25
530	6	30	1	25
531	6	31	1	-25
532	6	32	0	25
533	6	33	0	-25
534	6	34	1	-25
535	6	35	0	25
536	6	36	1	25
537	6	37	1	-25
538	6	38	0	25
539	6	39	1	-25
540	6	40	1	-25
541	6	41	0	25
542	6	42	0	-25
543	6	43	1	25
544	6	44	1	-25
545	6	45	1	25
546	6	46	0	25
547	6	47	1	-25
548	6	48	1	25
549	6	49	1	-25
550	6	50	0	25
551	6	51	1	0

Observation	Subject	Trial	Correct	Pressure
552	6	52	1	0
553	6	53	0	0
554	6	54	1	0
555	6	55	0	0
556	6	56	1	0
557	6	57	1	0
558	6	58	1	0
559	6	59	0	0
560	6	60	0	0
561	6	61	1	0
562	6	62	1	0
563	6	63	1	0
564	6	64	0	0
565	6	65	1	0
566	6	66	0	0
567	6	67	1	0
568	6	68	1	0
569	6	69	0	0
570	6	70	1	0
571	6	71	1	0
572	6	72	1	0
573	6	73	1	0
574	6	74	0	0
575	6	75	0	0
576	6	76	1	0
577	6	77	1	0
578	6	78	1	0
579	6	79	0	0
580	6	80	1	0
581	6	81	0	0
582	6	82	1	0
583	6	83	1	0
584	6	84	0	0
585	6	85	1	0
586	6	86	1	0
587	6	87	0	0
588	6	88	0	0
589	6	89	0	0
590	6	90	0	0
591	6	91	1	0
592	6	92	1	0
593	6	93	1	0
594	6	94	0	0
595	6	95	1	0

Observation	Subject	Trial	Correct	Pressure
596	6	96	0	0
597	6	97	0	0
598	6	98	0	0
599	6	99	0	0
600	6	100	0	0
601	7	1	1	-25
602	7	2	0	25
603	7	3	1	-25
604	7	4	1	-25
605	7	5	0	25
606	7	6	1	-25
607	7	7	1	25
608	7	8	0	-25
609	7	9	1	25
610	7	10	1	25
611	7	11	1	-25
612	7	12	0	25
613	7	13	0	-25
614	7	14	1	-25
615	7	15	1	25
616	7	16	0	-25
617	7	17	1	25
618	7	18	1	25
619	7	19	0	-25
620	7	20	0	-25
621	7	21	0	25
622	7	22	1	-25
623	7	23	1	25
624	7	24	1	-25
625	7	25	0	25
626	7	26	0	25
627	7	27	0	-25
628	7	28	1	25
629	7	29	0	-25
630	7	30	0	25
631	7	31	1	-25
632	7	32	0	25
633	7	33	0	-25
634	7	34	1	-25
635	7	35	0	25
636	7	36	1	25
637	7	37	1	-25
638	7	38	1	25
639	7	39	0	-25

Observation	Subject	Trial	Correct	Pressure
640	7	40	1	-25
641	7	41	1	25
642	7	42	0	-25
643	7	43	1	25
644	7	44	0	-25
645	7	45	0	25
646	7	46	0	25
647	7	47	1	-25
648	7	48	1	25
649	7	49	1	-25
650	7	50	0	25
651	7	51	0	0
652	7	52	1	0
653	7	53	1	0
654	7	54	1	0
655	7	55	0	0
656	7	56	1	0
657	7	57	1	0
658	7	58	0	0
659	7	59	0	0
660	7	60	1	0
661	7	61	1	0
662	7	62	0	0
663	7	63	1	0
664	7	64	0	0
665	7	65	0	0
666	7	66	0	0
667	7	67	1	0
668	7	68	0	0
669	7	69	1	0
670	7	70	1	0
671	7	71	1	0
672	7	72	1	0
673	7	73	0	0
674	7	74	1	0
675	7	75	0	0
676	7	76	0	0
677	7	77	0	0
678	7	78	1	0
679	7	79	0	0
680	7	80	1	0
681	7	81	0	0
682	7	82	1	0
683	7	83	1	0

Observation	Subject	Trial	Correct	Pressure
684	7	84	1	0
685	7	85	0	0
686	7	86	1	0
687	7	87	0	0
688	7	88	0	0
689	7	89	0	0
690	7	90	0	0
691	7	91	1	0
692	7	92	1	0
693	7	93	1	0
694	7	94	0	0
695	7	95	1	0
696	7	96	0	0
697	7	97	0	0
698	7	98	0	0
699	7	99	0	0
700	7	100	0	0
701	8	1	1	-25
702	8	2	1	25
703	8	3	1	-25
704	8	4	1	-25
705	8	5	0	25
706	8	6	1	-25
707	8	7	1	25
708	8	8	1	-25
709	8	9	1	25
710	8	10	1	25
711	8	11	1	-25
712	8	12	0	25
713	8	13	1	-25
714	8	14	0	-25
715	8	15	1	25
716	8	16	1	-25
717	8	17	0	25
718	8	18	1	25
719	8	19	1	-25
720	8	20	1	-25
721	8	21	1	25
722	8	22	1	-25
723	8	23	0	25
724	8	24	1	-25
725	8	25	1	25
726	8	26	0	25
727	8	27	1	-25

Observation	Subject	Trial	Correct	Pressure
728	8	28	1	25
729	8	29	0	-25
730	8	30	1	25
731	8	31	1	-25
732	8	32	0	25
733	8	33	0	-25
734	8	34	1	-25
735	8	35	0	25
736	8	36	0	25
737	8	37	1	-25
738	8	38	0	25
739	8	39	1	-25
740	8	40	1	-25
741	8	41	0	25
742	8	42	0	-25
743	8	43	1	25
744	8	44	1	-25
745	8	45	1	25
746	8	46	1	25
747	8	47	0	-25
748	8	48	1	25
749	8	49	1	-25
750	8	50	1	25
751	8	51	0	0
752	8	52	0	0
753	8	53	1	0
754	8	54	1	0
755	8	55	1	0
756	8	56	0	0
757	8	57	1	0
758	8	58	0	0
759	8	59	1	0
760	8	60	1	0
761	8	61	1	0
762	8	62	1	0
763	8	63	1	0
764	8	64	0	0
765	8	65	0	0
766	8	66	0	0
767	8	67	1	0
768	8	68	1	0
769	8	69	1	0
770	8	70	1	0
771	8	71	1	0

Observation	Subject	Trial	Correct	Pressure
772	8	72	1	0
773	8	73	1	0
774	8	74	1	0
775	8	75	1	0
776	8	76	0	0
777	8	77	1	0
778	8	78	1	0
779	8	79	0	0
780	8	80	1	0
781	8	81	1	0
782	8	82	1	0
783	8	83	1	0
784	8	84	1	0
785	8	85	1	0
786	8	86	1	0
787	8	87	0	0
788	8	88	1	0
789	8	89	1	0
790	8	90	0	0
791	8	91	1	0
792	8	92	0	0
793	8	93	1	0
794	8	94	0	0
795	8	95	1	0
796	8	96	1	0
797	8	97	1	0
798	8	98	1	0
799	8	99	1	0
800	8	100	0	0
801	9	1	1	0
802	9	2	1	0
803	9	3	1	0
804	9	4	1	0
805	9	5	1	0
806	9	6	1	0
807	9	7	0	0
808	9	8	1	0
809	9	9	1	0
810	9	10	1	0
811	9	11	1	0
812	9	12	1	0
813	9	13	1	0
814	9	14	1	0
815	9	15	1	0

Observation	Subject	Trial	Correct	Pressure
816	9	16	1	0
817	9	17	0	0
818	9	18	1	0
819	9	19	1	0
820	9	20	0	0
821	9	21	0	0
822	9	22	1	0
823	9	23	0	0
824	9	24	1	0
825	9	25	0	0
826	9	26	0	0
827	9	27	1	0
828	9	28	1	0
829	9	29	0	0
830	9	30	1	0
831	9	31	1	0
832	9	32	0	0
833	9	33	0	0
834	9	34	1	0
835	9	35	0	0
836	9	36	0	0
837	9	37	1	0
838	9	38	0	0
839	9	39	1	0
840	9	40	0	0
841	9	41	0	0
842	9	42	1	0
843	9	43	1	0
844	9	44	0	0
845	9	45	0	0
846	9	46	0	0
847	9	47	1	0
848	9	48	1	0
849	9	49	1	0
850	9	50	1	0
851	9	51	1	-25
852	9	52	0	25
853	9	53	1	-25
854	9	54	0	-25
855	9	55	0	25
856	9	56	0	-25
857	9	57	1	25
858	9	58	0	-25
859	9	59	0	25

Observation	Subject	Trial	Correct	Pressure
860	9	60	0	25
861	9	61	1	-25
862	9	62	1	25
863	9	63	1	-25
864	9	64	0	-25
865	9	65	1	25
866	9	66	0	-25
867	9	67	1	25
868	9	68	1	25
869	9	69	1	-25
870	9	70	1	-25
871	9	71	1	25
872	9	72	1	-25
873	9	73	0	25
874	9	74	1	-25
875	9	75	0	25
876	9	76	0	25
877	9	77	0	-25
878	9	78	1	25
879	9	79	0	-25
880	9	80	1	25
881	9	81	0	-25
882	9	82	1	25
883	9	83	1	-25
884	9	84	1	-25
885	9	85	1	25
886	9	86	1	25
887	9	87	0	-25
888	9	88	0	25
889	9	89	0	-25
890	9	90	0	-25
891	9	91	0	25
892	9	92	1	-25
893	9	93	1	25
894	9	94	0	-25
895	9	95	0	25
896	9	96	0	25
897	9	97	0	-25
898	9	98	1	25
899	9	99	0	-25
900	9	100	1	25
901	10	1	1	0
902	10	2	0	0
903	10	3	1	0

Observation	Subject	Trial	Correct	Pressure
904	10	4	1	0
905	10	5	0	0
906	10	6	1	0
907	10	7	1	0
908	10	8	0	0
909	10	9	1	0
910	10	10	1	0
911	10	11	1	0
912	10	12	1	0
913	10	13	1	0
914	10	14	0	0
915	10	15	1	0
916	10	16	1	0
917	10	17	1	0
918	10	18	1	0
919	10	19	1	0
920	10	20	0	0
921	10	21	1	0
922	10	22	1	0
923	10	23	0	0
924	10	24	1	0
925	10	25	0	0
926	10	26	1	0
927	10	27	0	0
928	10	28	1	0
929	10	29	0	0
930	10	30	1	0
931	10	31	1	0
932	10	32	1	0
933	10	33	1	0
934	10	34	1	0
935	10	35	1	0
936	10	36	0	0
937	10	37	1	0
938	10	38	0	0
939	10	39	1	0
940	10	40	1	0
941	10	41	1	0
942	10	42	0	0
943	10	43	1	0
944	10	44	0	0
945	10	45	1	0
946	10	46	1	0
947	10	47	1	0

Observation	Subject	Trial	Correct	Pressure
948	10	48	1	0
949	10	49	1	0
950	10	50	0	0
951	10	51	1	-25
952	10	52	1	25
953	10	53	1	-25
954	10	54	1	-25
955	10	55	0	25
956	10	56	0	-25
957	10	57	1	25
958	10	58	0	-25
959	10	59	1	25
960	10	60	0	25
961	10	61	1	-25
962	10	62	1	25
963	10	63	1	-25
964	10	64	0	-25
965	10	65	1	25
966	10	66	1	-25
967	10	67	0	25
968	10	68	0	25
969	10	69	1	-25
970	10	70	0	-25
971	10	71	1	25
972	10	72	0	-25
973	10	73	0	25
974	10	74	0	-25
975	10	75	1	25
976	10	76	0	25
977	10	77	1	-25
978	10	78	1	25
979	10	79	0	-25
980	10	80	1	25
981	10	81	0	-25
982	10	82	1	25
983	10	83	1	-25
984	10	84	1	-25
985	10	85	0	25
986	10	86	1	25
987	10	87	1	-25
988	10	88	0	25
989	10	89	0	-25
990	10	90	1	-25
991	10	91	0	25

Observation	Subject	Trial	Correct	Pressure
992	10	92	0	-25
993	10	93	1	25
994	10	94	0	-25
995	10	95	1	25
996	10	96	1	25
997	10	97	1	-25
998	10	98	1	25
999	10	99	0	-25
1000	10	100	0	25
1001	11	1	1	0
1002	11	2	0	0
1003	11	3	1	0
1004	11	4	1	0
1005	11	5	0	0
1006	11	6	1	0
1007	11	7	1	0
1008	11	8	0	0
1009	11	9	1	0
1010	11	10	1	0
1011	11	11	1	0
1012	11	12	0	0
1013	11	13	1	0
1014	11	14	0	0
1015	11	15	1	0
1016	11	16	1	0
1017	11	17	1	0
1018	11	18	1	0
1019	11	19	1	0
1020	11	20	0	0
1021	11	21	0	0
1022	11	22	1	0
1023	11	23	0	0
1024	11	24	1	0
1025	11	25	1	0
1026	11	26	1	0
1027	11	27	0	0
1028	11	28	1	0
1029	11	29	1	0
1030	11	30	0	0
1031	11	31	1	0
1032	11	32	0	0
1033	11	33	0	0
1034	11	34	1	0
1035	11	35	0	0

Observation	Subject	Trial	Correct	Pressure
1036	11	36	0	0
1037	11	37	1	0
1038	11	38	1	0
1039	11	39	1	0
1040	11	40	1	0
1041	11	41	0	0
1042	11	42	1	0
1043	11	43	1	0
1044	11	44	0	0
1045	11	45	1	0
1046	11	46	1	0
1047	11	47	1	0
1048	11	48	1	0
1049	11	49	1	0
1050	11	50	0	0
1051	11	51	0	-25
1052	11	52	0	25
1053	11	53	0	-25
1054	11	54	1	-25
1055	11	55	1	25
1056	11	56	1	-25
1057	11	57	1	25
1058	11	58	1	-25
1059	11	59	1	25
1060	11	60	1	25
1061	11	61	1	-25
1062	11	62	1	25
1063	11	63	1	-25
1064	11	64	0	-25
1065	11	65	1	25
1066	11	66	0	-25
1067	11	67	1	25
1068	11	68	1	25
1069	11	69	1	-25
1070	11	70	0	-25
1071	11	71	1	25
1072	11	72	0	-25
1073	11	73	1	25
1074	11	74	0	-25
1075	11	75	0	25
1076	11	76	0	25
1077	11	77	1	-25
1078	11	78	1	25
1079	11	79	0	-25

Observation	Subject	Trial	Correct	Pressure
1080	11	80	1	25
1081	11	81	0	-25
1082	11	82	1	25
1083	11	83	1	-25
1084	11	84	1	-25
1085	11	85	0	25
1086	11	86	1	25
1087	11	87	0	-25
1088	11	88	0	25
1089	11	89	1	-25
1090	11	90	1	-25
1091	11	91	0	25
1092	11	92	0	-25
1093	11	93	1	25
1094	11	94	1	-25
1095	11	95	1	25
1096	11	96	1	25
1097	11	97	0	-25
1098	11	98	0	25
1099	11	99	1	-25
1100	11	100	1	25
1101	12	1	1	0
1102	12	2	1	0
1103	12	3	1	0
1104	12	4	1	0
1105	12	5	0	0
1106	12	6	1	0
1107	12	7	1	0
1108	12	8	1	0
1109	12	9	1	0
1110	12	10	1	0
1111	12	11	1	0
1112	12	12	1	0
1113	12	13	1	0
1114	12	14	0	0
1115	12	15	1	0
1116	12	16	0	0
1117	12	17	0	0
1118	12	18	1	0
1119	12	19	1	0
1120	12	20	0	0
1121	12	21	0	0
1122	12	22	1	0
1123	12	23	0	0

Observation	Subject	Trial	Correct	Pressure
1124	12	24	1	0
1125	12	25	0	0
1126	12	26	1	0
1127	12	27	0	0
1128	12	28	1	0
1129	12	29	0	0
1130	12	30	0	0
1131	12	31	1	0
1132	12	32	0	0
1133	12	33	0	0
1134	12	34	1	0
1135	12	35	0	0
1136	12	36	0	0
1137	12	37	1	0
1138	12	38	0	0
1139	12	39	1	0
1140	12	40	1	0
1141	12	41	1	0
1142	12	42	1	0
1143	12	43	0	0
1144	12	44	0	0
1145	12	45	1	0
1146	12	46	0	0
1147	12	47	1	0
1148	12	48	1	0
1149	12	49	1	0
1150	12	50	0	0
1151	12	51	1	-25
1152	12	52	0	25
1153	12	53	1	-25
1154	12	54	1	-25
1155	12	55	0	25
1156	12	56	0	-25
1157	12	57	0	25
1158	12	58	1	-25
1159	12	59	0	25
1160	12	60	0	25
1161	12	61	1	-25
1162	12	62	1	25
1163	12	63	1	-25
1164	12	64	0	-25
1165	12	65	1	25
1166	12	66	0	-25
1167	12	67	1	25

Observation	Subject	Trial	Correct	Pressure
1168	12	68	0	25
1169	12	69	1	-25
1170	12	70	0	-25
1171	12	71	1	25
1172	12	72	0	-25
1173	12	73	0	25
1174	12	74	0	-25
1175	12	75	0	25
1176	12	76	0	25
1177	12	77	1	-25
1178	12	78	1	25
1179	12	79	1	-25
1180	12	80	1	25
1181	12	81	0	-25
1182	12	82	1	25
1183	12	83	1	-25
1184	12	84	0	-25
1185	12	85	1	25
1186	12	86	1	25
1187	12	87	0	-25
1188	12	88	1	25
1189	12	89	0	-25
1190	12	90	0	-25
1191	12	91	0	25
1192	12	92	0	-25
1193	12	93	1	25
1194	12	94	0	-25
1195	12	95	0	25
1196	12	96	1	25
1197	12	97	0	-25
1198	12	98	1	25
1199	12	99	0	-25
1200	12	100	0	25
1201	13	1	1	-25
1202	13	2	0	25
1203	13	3	0	-25
1204	13	4	1	-25
1205	13	5	0	25
1206	13	6	1	-25
1207	13	7	1	25
1208	13	8	1	-25
1209	13	9	1	25
1210	13	10	1	25
1211	13	11	1	-25

Observation	Subject	Trial	Correct	Pressure
1212	13	12	0	25
1213	13	13	0	-25
1214	13	14	1	-25
1215	13	15	1	25
1216	13	16	1	-25
1217	13	17	0	25
1218	13	18	1	25
1219	13	19	0	-25
1220	13	20	0	-25
1221	13	21	0	25
1222	13	22	1	-25
1223	13	23	0	25
1224	13	24	0	-25
1225	13	25	0	25
1226	13	26	1	25
1227	13	27	1	-25
1228	13	28	1	25
1229	13	29	1	-25
1230	13	30	1	25
1231	13	31	1	-25
1232	13	32	0	25
1233	13	33	0	-25
1234	13	34	1	-25
1235	13	35	1	25
1236	13	36	1	25
1237	13	37	1	-25
1238	13	38	0	25
1239	13	39	1	-25
1240	13	40	1	-25
1241	13	41	0	25
1242	13	42	1	-25
1243	13	43	1	25
1244	13	44	0	-25
1245	13	45	1	25
1246	13	46	1	25
1247	13	47	1	-25
1248	13	48	1	25
1249	13	49	0	-25
1250	13	50	0	25
1251	13	51	0	0
1252	13	52	0	0
1253	13	53	1	0
1254	13	54	1	0
1255	13	55	0	0

Observation	Subject	Trial	Correct	Pressure
1256	13	56	0	0
1257	13	57	1	0
1258	13	58	0	0
1259	13	59	1	0
1260	13	60	0	0
1261	13	61	1	0
1262	13	62	0	0
1263	13	63	1	0
1264	13	64	1	0
1265	13	65	1	0
1266	13	66	0	0
1267	13	67	0	0
1268	13	68	0	0
1269	13	69	1	0
1270	13	70	1	0
1271	13	71	1	0
1272	13	72	0	0
1273	13	73	0	0
1274	13	74	0	0
1275	13	75	0	0
1276	13	76	0	0
1277	13	77	1	0
1278	13	78	0	0
1279	13	79	1	0
1280	13	80	1	0
1281	13	81	0	0
1282	13	82	1	0
1283	13	83	1	0
1284	13	84	1	0
1285	13	85	1	0
1286	13	86	1	0
1287	13	87	0	0
1288	13	88	0	0
1289	13	89	0	0
1290	13	90	1	0
1291	13	91	0	0
1292	13	92	0	0
1293	13	93	1	0
1294	13	94	1	0
1295	13	95	1	0
1296	13	96	1	0
1297	13	97	0	0
1298	13	98	0	0
1299	13	99	1	0

Observation	Subject	Trial	Correct	Pressure
1300	13	100	1	0
1301	14	1	1	-25
1302	14	2	0	25
1303	14	3	0	-25
1304	14	4	1	-25
1305	14	5	0	25
1306	14	6	0	-25
1307	14	7	1	25
1308	14	8	0	-25
1309	14	9	1	25
1310	14	10	0	25
1311	14	11	1	-25
1312	14	12	1	25
1313	14	13	1	-25
1314	14	14	0	-25
1315	14	15	1	25
1316	14	16	1	-25
1317	14	17	1	25
1318	14	18	1	25
1319	14	19	1	-25
1320	14	20	1	-25
1321	14	21	0	25
1322	14	22	1	-25
1323	14	23	0	25
1324	14	24	0	-25
1325	14	25	0	25
1326	14	26	1	25
1327	14	27	0	-25
1328	14	28	1	25
1329	14	29	0	-25
1330	14	30	1	25
1331	14	31	1	-25
1332	14	32	0	25
1333	14	33	1	-25
1334	14	34	1	-25
1335	14	35	0	25
1336	14	36	0	25
1337	14	37	1	-25
1338	14	38	1	25
1339	14	39	0	-25
1340	14	40	1	-25
1341	14	41	1	25
1342	14	42	0	-25
1343	14	43	1	25

Observation	Subject	Trial	Correct	Pressure
1344	14	44	1	-25
1345	14	45	1	25
1346	14	46	1	25
1347	14	47	1	-25
1348	14	48	1	25
1349	14	49	0	-25
1350	14	50	1	25
1351	14	51	1	0
1352	14	52	0	0
1353	14	53	1	0
1354	14	54	1	0
1355	14	55	0	0
1356	14	56	1	0
1357	14	57	1	0
1358	14	58	0	0
1359	14	59	0	0
1360	14	60	0	0
1361	14	61	1	0
1362	14	62	1	0
1363	14	63	1	0
1364	14	64	1	0
1365	14	65	0	0
1366	14	66	0	0
1367	14	67	1	0
1368	14	68	1	0
1369	14	69	1	0
1370	14	70	0	0
1371	14	71	1	0
1372	14	72	0	0
1373	14	73	0	0
1374	14	74	0	0
1375	14	75	0	0
1376	14	76	0	0
1377	14	77	1	0
1378	14	78	1	0
1379	14	79	0	0
1380	14	80	1	0
1381	14	81	0	0
1382	14	82	1	0
1383	14	83	1	0
1384	14	84	1	0
1385	14	85	1	0
1386	14	86	1	0
1387	14	87	0	0

Observation	Subject	Trial	Correct	Pressure
1388	14	88	1	0
1389	14	89	1	0
1390	14	90	1	0
1391	14	91	0	0
1392	14	92	0	0
1393	14	93	1	0
1394	14	94	1	0
1395	14	95	1	0
1396	14	96	0	0
1397	14	97	1	0
1398	14	98	1	0
1399	14	99	1	0
1400	14	100	0	0
1401	15	1	0	-25
1402	15	2	1	25
1403	15	3	0	-25
1404	15	4	1	-25
1405	15	5	0	25
1406	15	6	1	-25
1407	15	7	1	25
1408	15	8	0	-25
1409	15	9	1	25
1410	15	10	1	25
1411	15	11	1	-25
1412	15	12	0	25
1413	15	13	0	-25
1414	15	14	0	-25
1415	15	15	1	25
1416	15	16	1	-25
1417	15	17	1	25
1418	15	18	0	25
1419	15	19	1	-25
1420	15	20	0	-25
1421	15	21	0	25
1422	15	22	1	-25
1423	15	23	1	25
1424	15	24	1	-25
1425	15	25	1	25
1426	15	26	0	25
1427	15	27	0	-25
1428	15	28	1	25
1429	15	29	0	-25
1430	15	30	0	25
1431	15	31	1	-25

Observation	Subject	Trial	Correct	Pressure
1432	15	32	1	25
1433	15	33	1	-25
1434	15	34	1	-25
1435	15	35	1	25
1436	15	36	0	25
1437	15	37	1	-25
1438	15	38	0	25
1439	15	39	0	-25
1440	15	40	1	-25
1441	15	41	0	25
1442	15	42	0	-25
1443	15	43	1	25
1444	15	44	0	-25
1445	15	45	1	25
1446	15	46	1	25
1447	15	47	1	-25
1448	15	48	1	25
1449	15	49	1	-25
1450	15	50	1	25
1451	15	51	1	0
1452	15	52	0	0
1453	15	53	1	0
1454	15	54	1	0
1455	15	55	0	0
1456	15	56	0	0
1457	15	57	1	0
1458	15	58	0	0
1459	15	59	0	0
1460	15	60	1	0
1461	15	61	1	0
1462	15	62	1	0
1463	15	63	1	0
1464	15	64	1	0
1465	15	65	0	0
1466	15	66	1	0
1467	15	67	1	0
1468	15	68	0	0
1469	15	69	1	0
1470	15	70	1	0
1471	15	71	1	0
1472	15	72	0	0
1473	15	73	0	0
1474	15	74	0	0
1475	15	75	0	0

Observation	Subject	Trial	Correct	Pressure
1476	15	76	0	0
1477	15	77	0	0
1478	15	78	1	0
1479	15	79	0	0
1480	15	80	1	0
1481	15	81	0	0
1482	15	82	1	0
1483	15	83	1	0
1484	15	84	1	0
1485	15	85	0	0
1486	15	86	1	0
1487	15	87	0	0
1488	15	88	0	0
1489	15	89	1	0
1490	15	90	1	0
1491	15	91	0	0
1492	15	92	1	0
1493	15	93	1	0
1494	15	94	0	0
1495	15	95	1	0
1496	15	96	1	0
1497	15	97	1	0
1498	15	98	0	0
1499	15	99	1	0
1500	15	100	0	0
1501	16	1	1	-25
1502	16	2	1	25
1503	16	3	1	-25
1504	16	4	1	-25
1505	16	5	0	25
1506	16	6	1	-25
1507	16	7	1	25
1508	16	8	0	-25
1509	16	9	1	25
1510	16	10	1	25
1511	16	11	1	-25
1512	16	12	1	25
1513	16	13	1	-25
1514	16	14	1	-25
1515	16	15	1	25
1516	16	16	1	-25
1517	16	17	0	25
1518	16	18	1	25
1519	16	19	1	-25

Observation	Subject	Trial	Correct	Pressure
1520	16	20	1	-25
1521	16	21	1	25
1522	16	22	1	-25
1523	16	23	0	25
1524	16	24	1	-25
1525	16	25	0	25
1526	16	26	0	25
1527	16	27	0	-25
1528	16	28	1	25
1529	16	29	1	-25
1530	16	30	1	25
1531	16	31	1	-25
1532	16	32	0	25
1533	16	33	1	-25
1534	16	34	1	-25
1535	16	35	1	25
1536	16	36	0	25
1537	16	37	1	-25
1538	16	38	1	25
1539	16	39	1	-25
1540	16	40	1	-25
1541	16	41	0	25
1542	16	42	0	-25
1543	16	43	0	25
1544	16	44	0	-25
1545	16	45	1	25
1546	16	46	0	25
1547	16	47	1	-25
1548	16	48	1	25
1549	16	49	1	-25
1550	16	50	0	25
1551	16	51	0	0
1552	16	52	1	0
1553	16	53	1	0
1554	16	54	1	0
1555	16	55	1	0
1556	16	56	0	0
1557	16	57	1	0
1558	16	58	0	0
1559	16	59	0	0
1560	16	60	0	0
1561	16	61	1	0
1562	16	62	1	0
1563	16	63	1	0

Observation	Subject	Trial	Correct	Pressure
1564	16	64	0	0
1565	16	65	1	0
1566	16	66	0	0
1567	16	67	1	0
1568	16	68	0	0
1569	16	69	1	0
1570	16	70	0	0
1571	16	71	1	0
1572	16	72	1	0
1573	16	73	1	0
1574	16	74	0	0
1575	16	75	0	0
1576	16	76	1	0
1577	16	77	1	0
1578	16	78	1	0
1579	16	79	1	0
1580	16	80	1	0
1581	16	81	0	0
1582	16	82	1	0
1583	16	83	1	0
1584	16	84	1	0
1585	16	85	0	0
1586	16	86	1	0
1587	16	87	0	0
1588	16	88	0	0
1589	16	89	1	0
1590	16	90	0	0
1591	16	91	1	0
1592	16	92	1	0
1593	16	93	1	0
1594	16	94	0	0
1595	16	95	1	0
1596	16	96	1	0
1597	16	97	1	0
1598	16	98	0	0
1599	16	99	1	0
1600	16	100	0	0
1601	17	1	1	0
1602	17	2	0	0
1603	17	3	1	0
1604	17	4	0	0
1605	17	5	0	0
1606	17	6	1	0
1607	17	7	1	0

Observation	Subject	Trial	Correct	Pressure
1608	17	8	0	0
1609	17	9	1	0
1610	17	10	1	0
1611	17	11	1	0
1612	17	12	0	0
1613	17	13	0	0
1614	17	14	1	0
1615	17	15	1	0
1616	17	16	1	0
1617	17	17	1	0
1618	17	18	1	0
1619	17	19	1	0
1620	17	20	1	0
1621	17	21	1	0
1622	17	22	0	0
1623	17	23	0	0
1624	17	24	0	0
1625	17	25	0	0
1626	17	26	0	0
1627	17	27	0	0
1628	17	28	1	0
1629	17	29	1	0
1630	17	30	0	0
1631	17	31	1	0
1632	17	32	0	0
1633	17	33	0	0
1634	17	34	1	0
1635	17	35	0	0
1636	17	36	0	0
1637	17	37	1	0
1638	17	38	1	0
1639	17	39	1	0
1640	17	40	0	0
1641	17	41	1	0
1642	17	42	0	0
1643	17	43	0	0
1644	17	44	0	0
1645	17	45	1	0
1646	17	46	1	0
1647	17	47	1	0
1648	17	48	1	0
1649	17	49	1	0
1650	17	50	0	0
1651	17	51	1	-25

Observation	Subject	Trial	Correct	Pressure
1652	17	52	0	25
1653	17	53	1	-25
1654	17	54	0	-25
1655	17	55	0	25
1656	17	56	0	-25
1657	17	57	1	25
1658	17	58	0	-25
1659	17	59	0	25
1660	17	60	0	25
1661	17	61	1	-25
1662	17	62	1	25
1663	17	63	1	-25
1664	17	64	0	-25
1665	17	65	1	25
1666	17	66	1	-25
1667	17	67	0	25
1668	17	68	0	25
1669	17	69	1	-25
1670	17	70	0	-25
1671	17	71	1	25
1672	17	72	1	-25
1673	17	73	1	25
1674	17	74	0	-25
1675	17	75	1	25
1676	17	76	0	25
1677	17	77	1	-25
1678	17	78	1	25
1679	17	79	0	-25
1680	17	80	1	25
1681	17	81	0	-25
1682	17	82	1	25
1683	17	83	1	-25
1684	17	84	0	-25
1685	17	85	0	25
1686	17	86	1	25
1687	17	87	0	-25
1688	17	88	0	25
1689	17	89	1	-25
1690	17	90	0	-25
1691	17	91	1	25
1692	17	92	0	-25
1693	17	93	1	25
1694	17	94	0	-25
1695	17	95	1	25

Observation	Subject	Trial	Correct	Pressure
1696	17	96	1	25
1697	17	97	0	-25
1698	17	98	1	25
1699	17	99	1	-25
1700	17	100	0	25
1701	18	1	1	0
1702	18	2	0	0
1703	18	3	1	0
1704	18	4	1	0
1705	18	5	1	0
1706	18	6	0	0
1707	18	7	1	0
1708	18	8	0	0
1709	18	9	1	0
1710	18	10	1	0
1711	18	11	1	0
1712	18	12	0	0
1713	18	13	1	0
1714	18	14	0	0
1715	18	15	1	0
1716	18	16	0	0
1717	18	17	1	0
1718	18	18	1	0
1719	18	19	1	0
1720	18	20	0	0
1721	18	21	1	0
1722	18	22	1	0
1723	18	23	0	0
1724	18	24	1	0
1725	18	25	1	0
1726	18	26	0	0
1727	18	27	1	0
1728	18	28	1	0
1729	18	29	0	0
1730	18	30	0	0
1731	18	31	1	0
1732	18	32	0	0
1733	18	33	0	0
1734	18	34	1	0
1735	18	35	0	0
1736	18	36	1	0
1737	18	37	1	0
1738	18	38	0	0
1739	18	39	1	0

Observation	Subject	Trial	Correct	Pressure
1740	18	40	0	0
1741	18	41	1	0
1742	18	42	0	0
1743	18	43	0	0
1744	18	44	0	0
1745	18	45	1	0
1746	18	46	0	0
1747	18	47	0	0
1748	18	48	1	0
1749	18	49	1	0
1750	18	50	1	0
1751	18	51	0	-25
1752	18	52	0	25
1753	18	53	1	-25
1754	18	54	1	-25
1755	18	55	0	25
1756	18	56	0	-25
1757	18	57	1	25
1758	18	58	1	-25
1759	18	59	0	25
1760	18	60	1	25
1761	18	61	1	-25
1762	18	62	1	25
1763	18	63	1	-25
1764	18	64	0	-25
1765	18	65	0	25
1766	18	66	0	-25
1767	18	67	0	25
1768	18	68	1	25
1769	18	69	1	-25
1770	18	70	0	-25
1771	18	71	1	25
1772	18	72	1	-25
1773	18	73	1	25
1774	18	74	0	-25
1775	18	75	0	25
1776	18	76	0	25
1777	18	77	1	-25
1778	18	78	1	25
1779	18	79	0	-25
1780	18	80	1	25
1781	18	81	0	-25
1782	18	82	1	25
1783	18	83	1	-25

Observation	Subject	Trial	Correct	Pressure
1784	18	84	1	-25
1785	18	85	0	25
1786	18	86	1	25
1787	18	87	0	-25
1788	18	88	1	25
1789	18	89	1	-25
1790	18	90	0	-25
1791	18	91	0	25
1792	18	92	0	-25
1793	18	93	1	25
1794	18	94	0	-25
1795	18	95	1	25
1796	18	96	1	25
1797	18	97	0	-25
1798	18	98	0	25
1799	18	99	1	-25
1800	18	100	1	25
1801	19	1	1	0
1802	19	2	1	0
1803	19	3	1	0
1804	19	4	0	0
1805	19	5	0	0
1806	19	6	1	0
1807	19	7	1	0
1808	19	8	0	0
1809	19	9	1	0
1810	19	10	1	0
1811	19	11	1	0
1812	19	12	0	0
1813	19	13	1	0
1814	19	14	0	0
1815	19	15	1	0
1816	19	16	0	0
1817	19	17	1	0
1818	19	18	1	0
1819	19	19	1	0
1820	19	20	1	0
1821	19	21	1	0
1822	19	22	1	0
1823	19	23	0	0
1824	19	24	1	0
1825	19	25	0	0
1826	19	26	0	0
1827	19	27	1	0

Observation	Subject	Trial	Correct	Pressure
1828	19	28	1	0
1829	19	29	1	0
1830	19	30	0	0
1831	19	31	1	0
1832	19	32	0	0
1833	19	33	0	0
1834	19	34	1	0
1835	19	35	1	0
1836	19	36	0	0
1837	19	37	1	0
1838	19	38	0	0
1839	19	39	1	0
1840	19	40	1	0
1841	19	41	0	0
1842	19	42	1	0
1843	19	43	1	0
1844	19	44	1	0
1845	19	45	1	0
1846	19	46	1	0
1847	19	47	1	0
1848	19	48	1	0
1849	19	49	1	0
1850	19	50	0	0
1851	19	51	1	-25
1852	19	52	1	25
1853	19	53	1	-25
1854	19	54	0	-25
1855	19	55	1	25
1856	19	56	0	-25
1857	19	57	1	25
1858	19	58	1	-25
1859	19	59	0	25
1860	19	60	1	25
1861	19	61	1	-25
1862	19	62	1	25
1863	19	63	1	-25
1864	19	64	1	-25
1865	19	65	1	25
1866	19	66	0	-25
1867	19	67	1	25
1868	19	68	1	25
1869	19	69	1	-25
1870	19	70	0	-25
1871	19	71	0	25

Observation	Subject	Trial	Correct	Pressure
1872	19	72	1	-25
1873	19	73	1	25
1874	19	74	0	-25
1875	19	75	0	25
1876	19	76	0	25
1877	19	77	1	-25
1878	19	78	1	25
1879	19	79	0	-25
1880	19	80	1	25
1881	19	81	0	-25
1882	19	82	1	25
1883	19	83	1	-25
1884	19	84	1	-25
1885	19	85	1	25
1886	19	86	1	25
1887	19	87	0	-25
1888	19	88	1	25
1889	19	89	0	-25
1890	19	90	0	-25
1891	19	91	0	25
1892	19	92	0	-25
1893	19	93	1	25
1894	19	94	0	-25
1895	19	95	1	25
1896	19	96	1	25
1897	19	97	0	-25
1898	19	98	0	25
1899	19	99	1	-25
1900	19	100	0	25
1901	20	1	1	0
1902	20	2	1	0
1903	20	3	1	0
1904	20	4	1	0
1905	20	5	0	0
1906	20	6	1	0
1907	20	7	1	0
1908	20	8	1	0
1909	20	9	1	0
1910	20	10	1	0
1911	20	11	1	0
1912	20	12	1	0
1913	20	13	1	0
1914	20	14	0	0
1915	20	15	1	0

Observation	Subject	Trial	Correct	Pressure
1916	20	16	1	0
1917	20	17	1	0
1918	20	18	1	0
1919	20	19	1	0
1920	20	20	0	0
1921	20	21	0	0
1922	20	22	1	0
1923	20	23	1	0
1924	20	24	0	0
1925	20	25	0	0
1926	20	26	1	0
1927	20	27	0	0
1928	20	28	1	0
1929	20	29	0	0
1930	20	30	1	0
1931	20	31	1	0
1932	20	32	0	0
1933	20	33	0	0
1934	20	34	1	0
1935	20	35	1	0
1936	20	36	1	0
1937	20	37	1	0
1938	20	38	0	0
1939	20	39	1	0
1940	20	40	1	0
1941	20	41	1	0
1942	20	42	1	0
1943	20	43	1	0
1944	20	44	1	0
1945	20	45	0	0
1946	20	46	1	0
1947	20	47	1	0
1948	20	48	1	0
1949	20	49	1	0
1950	20	50	0	0
1951	20	51	1	-25
1952	20	52	1	25
1953	20	53	1	-25
1954	20	54	1	-25
1955	20	55	0	25
1956	20	56	0	-25
1957	20	57	1	25
1958	20	58	0	-25
1959	20	59	0	25

Observation	Subject	Trial	Correct	Pressure
1960	20	60	1	25
1961	20	61	1	-25
1962	20	62	1	25
1963	20	63	1	-25
1964	20	64	0	-25
1965	20	65	0	25
1966	20	66	1	-25
1967	20	67	0	25
1968	20	68	0	25
1969	20	69	1	-25
1970	20	70	0	-25
1971	20	71	1	25
1972	20	72	1	-25
1973	20	73	1	25
1974	20	74	1	-25
1975	20	75	0	25
1976	20	76	1	25
1977	20	77	1	-25
1978	20	78	0	25
1979	20	79	0	-25
1980	20	80	1	25
1981	20	81	1	-25
1982	20	82	1	25
1983	20	83	1	-25
1984	20	84	1	-25
1985	20	85	0	25
1986	20	86	1	25
1987	20	87	0	-25
1988	20	88	1	25
1989	20	89	0	-25
1990	20	90	0	-25
1991	20	91	0	25
1992	20	92	1	-25
1993	20	93	1	25
1994	20	94	0	-25
1995	20	95	1	25
1996	20	96	1	25
1997	20	97	1	-25
1998	20	98	1	25
1999	20	99	0	-25
2000	20	100	1	25
2001	21	1	1	-25
2002	21	2	0	25
2003	21	3	0	-25

Observation	Subject	Trial	Correct	Pressure
2004	21	4	1	-25
2005	21	5	0	25
2006	21	6	1	-25
2007	21	7	1	25
2008	21	8	0	-25
2009	21	9	1	25
2010	21	10	1	25
2011	21	11	0	-25
2012	21	12	0	25
2013	21	13	1	-25
2014	21	14	0	-25
2015	21	15	1	25
2016	21	16	0	-25
2017	21	17	1	25
2018	21	18	0	25
2019	21	19	1	-25
2020	21	20	0	-25
2021	21	21	0	25
2022	21	22	1	-25
2023	21	23	1	25
2024	21	24	1	-25
2025	21	25	0	25
2026	21	26	1	25
2027	21	27	0	-25
2028	21	28	1	25
2029	21	29	0	-25
2030	21	30	0	25
2031	21	31	1	-25
2032	21	32	1	25
2033	21	33	0	-25
2034	21	34	1	-25
2035	21	35	1	25
2036	21	36	1	25
2037	21	37	1	-25
2038	21	38	0	25
2039	21	39	1	-25
2040	21	40	0	-25
2041	21	41	0	25
2042	21	42	0	-25
2043	21	43	1	25
2044	21	44	0	-25
2045	21	45	1	25
2046	21	46	0	25
2047	21	47	1	-25

Observation	Subject	Trial	Correct	Pressure
2048	21	48	1	25
2049	21	49	1	-25
2050	21	50	0	25
2051	21	51	0	0
2052	21	52	0	0
2053	21	53	1	0
2054	21	54	1	0
2055	21	55	0	0
2056	21	56	0	0
2057	21	57	1	0
2058	21	58	1	0
2059	21	59	1	0
2060	21	60	0	0
2061	21	61	1	0
2062	21	62	1	0
2063	21	63	1	0
2064	21	64	0	0
2065	21	65	1	0
2066	21	66	0	0
2067	21	67	1	0
2068	21	68	1	0
2069	21	69	1	0
2070	21	70	1	0
2071	21	71	1	0
2072	21	72	0	0
2073	21	73	0	0
2074	21	74	0	0
2075	21	75	0	0
2076	21	76	1	0
2077	21	77	0	0
2078	21	78	1	0
2079	21	79	0	0
2080	21	80	1	0
2081	21	81	1	0
2082	21	82	1	0
2083	21	83	1	0
2084	21	84	1	0
2085	21	85	0	0
2086	21	86	1	0
2087	21	87	0	0
2088	21	88	0	0
2089	21	89	1	0
2090	21	90	0	0
2091	21	91	0	0

Observation	Subject	Trial	Correct	Pressure
2092	21	92	0	0
2093	21	93	1	0
2094	21	94	0	0
2095	21	95	1	0
2096	21	96	1	0
2097	21	97	1	0
2098	21	98	0	0
2099	21	99	1	0
2100	21	100	0	0
2101	22	1	1	-25
2102	22	2	0	25
2103	22	3	1	-25
2104	22	4	0	-25
2105	22	5	0	25
2106	22	6	1	-25
2107	22	7	1	25
2108	22	8	1	-25
2109	22	9	1	25
2110	22	10	1	25
2111	22	11	1	-25
2112	22	12	1	25
2113	22	13	0	-25
2114	22	14	0	-25
2115	22	15	1	25
2116	22	16	0	-25
2117	22	17	0	25
2118	22	18	1	25
2119	22	19	0	-25
2120	22	20	0	-25
2121	22	21	1	25
2122	22	22	1	-25
2123	22	23	1	25
2124	22	24	1	-25
2125	22	25	1	25
2126	22	26	1	25
2127	22	27	0	-25
2128	22	28	1	25
2129	22	29	0	-25
2130	22	30	1	25
2131	22	31	1	-25
2132	22	32	1	25
2133	22	33	0	-25
2134	22	34	1	-25
2135	22	35	0	25

Observation	Subject	Trial	Correct	Pressure
2136	22	36	0	25
2137	22	37	1	-25
2138	22	38	0	25
2139	22	39	0	-25
2140	22	40	1	-25
2141	22	41	1	25
2142	22	42	1	-25
2143	22	43	1	25
2144	22	44	0	-25
2145	22	45	1	25
2146	22	46	0	25
2147	22	47	0	-25
2148	22	48	0	25
2149	22	49	0	-25
2150	22	50	0	25
2151	22	51	0	0
2152	22	52	1	0
2153	22	53	0	0
2154	22	54	0	0
2155	22	55	0	0
2156	22	56	1	0
2157	22	57	1	0
2158	22	58	0	0
2159	22	59	0	0
2160	22	60	0	0
2161	22	61	1	0
2162	22	62	1	0
2163	22	63	1	0
2164	22	64	0	0
2165	22	65	0	0
2166	22	66	0	0
2167	22	67	0	0
2168	22	68	0	0
2169	22	69	1	0
2170	22	70	1	0
2171	22	71	1	0
2172	22	72	0	0
2173	22	73	1	0
2174	22	74	1	0
2175	22	75	1	0
2176	22	76	0	0
2177	22	77	1	0
2178	22	78	1	0
2179	22	79	0	0

Observation	Subject	Trial	Correct	Pressure
2180	22	80	1	0
2181	22	81	1	0
2182	22	82	1	0
2183	22	83	1	0
2184	22	84	1	0
2185	22	85	0	0
2186	22	86	1	0
2187	22	87	0	0
2188	22	88	1	0
2189	22	89	0	0
2190	22	90	0	0
2191	22	91	0	0
2192	22	92	0	0
2193	22	93	1	0
2194	22	94	1	0
2195	22	95	1	0
2196	22	96	0	0
2197	22	97	0	0
2198	22	98	1	0
2199	22	99	1	0
2200	22	100	0	0
2201	23	1	1	-25
2202	23	2	0	25
2203	23	3	1	-25
2204	23	4	1	-25
2205	23	5	0	25
2206	23	6	1	-25
2207	23	7	1	25
2208	23	8	0	-25
2209	23	9	1	25
2210	23	10	1	25
2211	23	11	1	-25
2212	23	12	0	25
2213	23	13	0	-25
2214	23	14	1	-25
2215	23	15	1	25
2216	23	16	0	-25
2217	23	17	0	25
2218	23	18	1	25
2219	23	19	1	-25
2220	23	20	0	-25
2221	23	21	0	25
2222	23	22	1	-25
2223	23	23	0	25

Observation	Subject	Trial	Correct	Pressure
2224	23	24	1	-25
2225	23	25	0	25
2226	23	26	1	25
2227	23	27	0	-25
2228	23	28	1	25
2229	23	29	1	-25
2230	23	30	0	25
2231	23	31	1	-25
2232	23	32	0	25
2233	23	33	0	-25
2234	23	34	1	-25
2235	23	35	1	25
2236	23	36	0	25
2237	23	37	1	-25
2238	23	38	0	25
2239	23	39	0	-25
2240	23	40	1	-25
2241	23	41	1	25
2242	23	42	1	-25
2243	23	43	0	25
2244	23	44	0	-25
2245	23	45	1	25
2246	23	46	0	25
2247	23	47	1	-25
2248	23	48	1	25
2249	23	49	1	-25
2250	23	50	0	25
2251	23	51	0	0
2252	23	52	1	0
2253	23	53	1	0
2254	23	54	0	0
2255	23	55	0	0
2256	23	56	0	0
2257	23	57	1	0
2258	23	58	1	0
2259	23	59	0	0
2260	23	60	0	0
2261	23	61	1	0
2262	23	62	1	0
2263	23	63	1	0
2264	23	64	0	0
2265	23	65	1	0
2266	23	66	0	0
2267	23	67	1	0

Observation	Subject	Trial	Correct	Pressure
2268	23	68	0	0
2269	23	69	1	0
2270	23	70	1	0
2271	23	71	1	0
2272	23	72	0	0
2273	23	73	0	0
2274	23	74	0	0
2275	23	75	1	0
2276	23	76	0	0
2277	23	77	1	0
2278	23	78	0	0
2279	23	79	1	0
2280	23	80	1	0
2281	23	81	1	0
2282	23	82	1	0
2283	23	83	1	0
2284	23	84	1	0
2285	23	85	0	0
2286	23	86	1	0
2287	23	87	1	0
2288	23	88	1	0
2289	23	89	1	0
2290	23	90	0	0
2291	23	91	0	0
2292	23	92	0	0
2293	23	93	1	0
2294	23	94	0	0
2295	23	95	1	0
2296	23	96	1	0
2297	23	97	1	0
2298	23	98	0	0
2299	23	99	0	0
2300	23	100	0	0
2301	24	1	1	-25
2302	24	2	0	25
2303	24	3	1	-25
2304	24	4	1	-25
2305	24	5	1	25
2306	24	6	1	-25
2307	24	7	0	25
2308	24	8	1	-25
2309	24	9	1	25
2310	24	10	1	25
2311	24	11	1	-25

Observation	Subject	Trial	Correct	Pressure
2312	24	12	0	25
2313	24	13	0	-25
2314	24	14	0	-25
2315	24	15	1	25
2316	24	16	1	-25
2317	24	17	0	25
2318	24	18	1	25
2319	24	19	1	-25
2320	24	20	1	-25
2321	24	21	0	25
2322	24	22	1	-25
2323	24	23	1	25
2324	24	24	1	-25
2325	24	25	0	25
2326	24	26	0	25
2327	24	27	0	-25
2328	24	28	1	25
2329	24	29	0	-25
2330	24	30	0	25
2331	24	31	1	-25
2332	24	32	0	25
2333	24	33	0	-25
2334	24	34	1	-25
2335	24	35	0	25
2336	24	36	1	25
2337	24	37	1	-25
2338	24	38	1	25
2339	24	39	1	-25
2340	24	40	1	-25
2341	24	41	1	25
2342	24	42	0	-25
2343	24	43	1	25
2344	24	44	1	-25
2345	24	45	1	25
2346	24	46	0	25
2347	24	47	0	-25
2348	24	48	1	25
2349	24	49	1	-25
2350	24	50	0	25
2351	24	51	1	0
2352	24	52	0	0
2353	24	53	1	0
2354	24	54	1	0
2355	24	55	0	0

Observation	Subject	Trial	Correct	Pressure
2356	24	56	1	0
2357	24	57	1	0
2358	24	58	0	0
2359	24	59	1	0
2360	24	60	0	0
2361	24	61	1	0
2362	24	62	1	0
2363	24	63	1	0
2364	24	64	1	0
2365	24	65	1	0
2366	24	66	0	0
2367	24	67	0	0
2368	24	68	0	0
2369	24	69	1	0
2370	24	70	0	0
2371	24	71	0	0
2372	24	72	0	0
2373	24	73	0	0
2374	24	74	0	0
2375	24	75	0	0
2376	24	76	0	0
2377	24	77	1	0
2378	24	78	1	0
2379	24	79	0	0
2380	24	80	0	0
2381	24	81	0	0
2382	24	82	1	0
2383	24	83	1	0
2384	24	84	1	0
2385	24	85	0	0
2386	24	86	1	0
2387	24	87	1	0
2388	24	88	0	0
2389	24	89	0	0
2390	24	90	0	0
2391	24	91	0	0
2392	24	92	0	0
2393	24	93	1	0
2394	24	94	0	0
2395	24	95	1	0
2396	24	96	1	0
2397	24	97	0	0
2398	24	98	1	0
2399	24	99	1	0

Observation	Subject	Trial	Correct	Pressure
2400	24	100	0	0
2401	25	1	1	0
2402	25	2	1	0
2403	25	3	0	0
2404	25	4	1	0
2405	25	5	0	0
2406	25	6	1	0
2407	25	7	1	0
2408	25	8	0	0
2409	25	9	1	0
2410	25	10	1	0
2411	25	11	1	0
2412	25	12	1	0
2413	25	13	0	0
2414	25	14	0	0
2415	25	15	1	0
2416	25	16	0	0
2417	25	17	1	0
2418	25	18	1	0
2419	25	19	1	0
2420	25	20	1	0
2421	25	21	1	0
2422	25	22	1	0
2423	25	23	0	0
2424	25	24	1	0
2425	25	25	0	0
2426	25	26	0	0
2427	25	27	0	0
2428	25	28	1	0
2429	25	29	0	0
2430	25	30	0	0
2431	25	31	1	0
2432	25	32	0	0
2433	25	33	0	0
2434	25	34	1	0
2435	25	35	0	0
2436	25	36	0	0
2437	25	37	1	0
2438	25	38	0	0
2439	25	39	1	0
2440	25	40	0	0
2441	25	41	1	0
2442	25	42	1	0
2443	25	43	1	0

Observation	Subject	Trial	Correct	Pressure
2444	25	44	1	0
2445	25	45	0	0
2446	25	46	0	0
2447	25	47	1	0
2448	25	48	1	0
2449	25	49	1	0
2450	25	50	1	0
2451	25	51	0	-25
2452	25	52	0	25
2453	25	53	1	-25
2454	25	54	0	-25
2455	25	55	0	25
2456	25	56	1	-25
2457	25	57	1	25
2458	25	58	0	-25
2459	25	59	1	25
2460	25	60	0	25
2461	25	61	1	-25
2462	25	62	1	25
2463	25	63	1	-25
2464	25	64	1	-25
2465	25	65	0	25
2466	25	66	0	-25
2467	25	67	0	25
2468	25	68	0	25
2469	25	69	1	-25
2470	25	70	0	-25
2471	25	71	1	25
2472	25	72	0	-25
2473	25	73	0	25
2474	25	74	0	-25
2475	25	75	0	25
2476	25	76	0	25
2477	25	77	1	-25
2478	25	78	0	25
2479	25	79	0	-25
2480	25	80	1	25
2481	25	81	0	-25
2482	25	82	1	25
2483	25	83	1	-25
2484	25	84	1	-25
2485	25	85	0	25
2486	25	86	1	25
2487	25	87	0	-25

Observation	Subject	Trial	Correct	Pressure
2488	25	88	1	25
2489	25	89	0	-25
2490	25	90	0	-25
2491	25	91	0	25
2492	25	92	0	-25
2493	25	93	1	25
2494	25	94	0	-25
2495	25	95	1	25
2496	25	96	1	25
2497	25	97	0	-25
2498	25	98	0	25
2499	25	99	0	-25
2500	25	100	0	25
2501	26	1	1	0
2502	26	2	1	0
2503	26	3	1	0
2504	26	4	1	0
2505	26	5	0	0
2506	26	6	1	0
2507	26	7	1	0
2508	26	8	1	0
2509	26	9	0	0
2510	26	10	1	0
2511	26	11	1	0
2512	26	12	1	0
2513	26	13	1	0
2514	26	14	0	0
2515	26	15	1	0
2516	26	16	1	0
2517	26	17	1	0
2518	26	18	1	0
2519	26	19	1	0
2520	26	20	1	0
2521	26	21	1	0
2522	26	22	1	0
2523	26	23	0	0
2524	26	24	1	0
2525	26	25	1	0
2526	26	26	1	0
2527	26	27	0	0
2528	26	28	1	0
2529	26	29	0	0
2530	26	30	0	0
2531	26	31	0	0

Observation	Subject	Trial	Correct	Pressure
2532	26	32	1	0
2533	26	33	0	0
2534	26	34	1	0
2535	26	35	1	0
2536	26	36	1	0
2537	26	37	0	0
2538	26	38	0	0
2539	26	39	1	0
2540	26	40	0	0
2541	26	41	0	0
2542	26	42	1	0
2543	26	43	0	0
2544	26	44	1	0
2545	26	45	1	0
2546	26	46	1	0
2547	26	47	1	0
2548	26	48	1	0
2549	26	49	0	0
2550	26	50	0	0
2551	26	51	0	-25
2552	26	52	1	25
2553	26	53	1	-25
2554	26	54	0	-25
2555	26	55	0	25
2556	26	56	1	-25
2557	26	57	1	25
2558	26	58	1	-25
2559	26	59	1	25
2560	26	60	0	25
2561	26	61	1	-25
2562	26	62	1	25
2563	26	63	1	-25
2564	26	64	0	-25
2565	26	65	0	25
2566	26	66	1	-25
2567	26	67	0	25
2568	26	68	1	25
2569	26	69	1	-25
2570	26	70	1	-25
2571	26	71	1	25
2572	26	72	0	-25
2573	26	73	1	25
2574	26	74	0	-25
2575	26	75	0	25

Observation	Subject	Trial	Correct	Pressure
2576	26	76	0	25
2577	26	77	1	-25
2578	26	78	1	25
2579	26	79	0	-25
2580	26	80	1	25
2581	26	81	0	-25
2582	26	82	1	25
2583	26	83	1	-25
2584	26	84	1	-25
2585	26	85	0	25
2586	26	86	1	25
2587	26	87	0	-25
2588	26	88	1	25
2589	26	89	1	-25
2590	26	90	1	-25
2591	26	91	1	25
2592	26	92	1	-25
2593	26	93	1	25
2594	26	94	0	-25
2595	26	95	1	25
2596	26	96	1	25
2597	26	97	1	-25
2598	26	98	1	25
2599	26	99	1	-25
2600	26	100	1	25
2601	27	1	1	0
2602	27	2	1	0
2603	27	3	1	0
2604	27	4	1	0
2605	27	5	0	0
2606	27	6	0	0
2607	27	7	1	0
2608	27	8	0	0
2609	27	9	1	0
2610	27	10	1	0
2611	27	11	1	0
2612	27	12	1	0
2613	27	13	0	0
2614	27	14	1	0
2615	27	15	1	0
2616	27	16	1	0
2617	27	17	0	0
2618	27	18	1	0
2619	27	19	0	0

Observation	Subject	Trial	Correct	Pressure
2620	27	20	0	0
2621	27	21	0	0
2622	27	22	1	0
2623	27	23	0	0
2624	27	24	0	0
2625	27	25	0	0
2626	27	26	0	0
2627	27	27	1	0
2628	27	28	1	0
2629	27	29	0	0
2630	27	30	0	0
2631	27	31	0	0
2632	27	32	1	0
2633	27	33	1	0
2634	27	34	1	0
2635	27	35	0	0
2636	27	36	0	0
2637	27	37	1	0
2638	27	38	0	0
2639	27	39	1	0
2640	27	40	0	0
2641	27	41	0	0
2642	27	42	1	0
2643	27	43	1	0
2644	27	44	1	0
2645	27	45	1	0
2646	27	46	0	0
2647	27	47	0	0
2648	27	48	1	0
2649	27	49	1	0
2650	27	50	0	0
2651	27	51	0	-25
2652	27	52	1	25
2653	27	53	1	-25
2654	27	54	1	-25
2655	27	55	0	25
2656	27	56	1	-25
2657	27	57	1	25
2658	27	58	0	-25
2659	27	59	1	25
2660	27	60	0	25
2661	27	61	1	-25
2662	27	62	1	25
2663	27	63	1	-25

Observation	Subject	Trial	Correct	Pressure
2664	27	64	0	-25
2665	27	65	0	25
2666	27	66	0	-25
2667	27	67	1	25
2668	27	68	1	25
2669	27	69	1	-25
2670	27	70	0	-25
2671	27	71	1	25
2672	27	72	0	-25
2673	27	73	1	25
2674	27	74	0	-25
2675	27	75	0	25
2676	27	76	0	25
2677	27	77	1	-25
2678	27	78	1	25
2679	27	79	0	-25
2680	27	80	1	25
2681	27	81	0	-25
2682	27	82	1	25
2683	27	83	1	-25
2684	27	84	1	-25
2685	27	85	0	25
2686	27	86	1	25
2687	27	87	0	-25
2688	27	88	1	25
2689	27	89	0	-25
2690	27	90	0	-25
2691	27	91	0	25
2692	27	92	0	-25
2693	27	93	1	25
2694	27	94	0	-25
2695	27	95	0	25
2696	27	96	1	25
2697	27	97	1	-25
2698	27	98	0	25
2699	27	99	0	-25
2700	27	100	1	25
2701	28	1	1	0
2702	28	2	0	0
2703	28	3	1	0
2704	28	4	1	0
2705	28	5	0	0
2706	28	6	0	0
2707	28	7	1	0

Observation	Subject	Trial	Correct	Pressure
2708	28	8	0	0
2709	28	9	1	0
2710	28	10	1	0
2711	28	11	1	0
2712	28	12	0	0
2713	28	13	0	0
2714	28	14	0	0
2715	28	15	1	0
2716	28	16	0	0
2717	28	17	0	0
2718	28	18	1	0
2719	28	19	0	0
2720	28	20	1	0
2721	28	21	1	0
2722	28	22	1	0
2723	28	23	1	0
2724	28	24	1	0
2725	28	25	0	0
2726	28	26	1	0
2727	28	27	1	0
2728	28	28	1	0
2729	28	29	1	0
2730	28	30	0	0
2731	28	31	1	0
2732	28	32	0	0
2733	28	33	0	0
2734	28	34	1	0
2735	28	35	0	0
2736	28	36	1	0
2737	28	37	0	0
2738	28	38	0	0
2739	28	39	1	0
2740	28	40	1	0
2741	28	41	1	0
2742	28	42	0	0
2743	28	43	0	0
2744	28	44	0	0
2745	28	45	1	0
2746	28	46	0	0
2747	28	47	0	0
2748	28	48	1	0
2749	28	49	0	0
2750	28	50	0	0
2751	28	51	0	-25

Observation	Subject	Trial	Correct	Pressure
2752	28	52	0	25
2753	28	53	1	-25
2754	28	54	1	-25
2755	28	55	0	25
2756	28	56	0	-25
2757	28	57	1	25
2758	28	58	0	-25
2759	28	59	0	25
2760	28	60	0	25
2761	28	61	1	-25
2762	28	62	1	25
2763	28	63	1	-25
2764	28	64	0	-25
2765	28	65	0	25
2766	28	66	0	-25
2767	28	67	0	25
2768	28	68	1	25
2769	28	69	1	-25
2770	28	70	1	-25
2771	28	71	1	25
2772	28	72	0	-25
2773	28	73	0	25
2774	28	74	0	-25
2775	28	75	0	25
2776	28	76	0	25
2777	28	77	1	-25
2778	28	78	1	25
2779	28	79	1	-25
2780	28	80	1	25
2781	28	81	0	-25
2782	28	82	1	25
2783	28	83	1	-25
2784	28	84	1	-25
2785	28	85	0	25
2786	28	86	1	25
2787	28	87	0	-25
2788	28	88	0	25
2789	28	89	0	-25
2790	28	90	0	-25
2791	28	91	1	25
2792	28	92	0	-25
2793	28	93	1	25
2794	28	94	0	-25
2795	28	95	1	25

Observation	Subject	Trial	Correct	Pressure
2796	28	96	1	25
2797	28	97	0	-25
2798	28	98	0	25
2799	28	99	1	-25
2800	28	100	1	25
2801	29	1	1	-25
2802	29	2	1	25
2803	29	3	0	-25
2804	29	4	1	-25
2805	29	5	0	25
2806	29	6	1	-25
2807	29	7	1	25
2808	29	8	0	-25
2809	29	9	0	25
2810	29	10	1	25
2811	29	11	0	-25
2812	29	12	1	25
2813	29	13	0	-25
2814	29	14	0	-25
2815	29	15	1	25
2816	29	16	0	-25
2817	29	17	0	25
2818	29	18	1	25
2819	29	19	1	-25
2820	29	20	0	-25
2821	29	21	1	25
2822	29	22	1	-25
2823	29	23	1	25
2824	29	24	1	-25
2825	29	25	1	25
2826	29	26	0	25
2827	29	27	0	-25
2828	29	28	1	25
2829	29	29	1	-25
2830	29	30	0	25
2831	29	31	1	-25
2832	29	32	0	25
2833	29	33	0	-25
2834	29	34	1	-25
2835	29	35	0	25
2836	29	36	0	25
2837	29	37	1	-25
2838	29	38	0	25
2839	29	39	1	-25

Observation	Subject	Trial	Correct	Pressure
2840	29	40	1	-25
2841	29	41	1	25
2842	29	42	1	-25
2843	29	43	1	25
2844	29	44	1	-25
2845	29	45	1	25
2846	29	46	0	25
2847	29	47	1	-25
2848	29	48	1	25
2849	29	49	1	-25
2850	29	50	0	25
2851	29	51	1	0
2852	29	52	1	0
2853	29	53	1	0
2854	29	54	1	0
2855	29	55	1	0
2856	29	56	0	0
2857	29	57	1	0
2858	29	58	0	0
2859	29	59	0	0
2860	29	60	0	0
2861	29	61	1	0
2862	29	62	1	0
2863	29	63	1	0
2864	29	64	0	0
2865	29	65	1	0
2866	29	66	0	0
2867	29	67	0	0
2868	29	68	1	0
2869	29	69	1	0
2870	29	70	0	0
2871	29	71	1	0
2872	29	72	1	0
2873	29	73	1	0
2874	29	74	0	0
2875	29	75	0	0
2876	29	76	0	0
2877	29	77	1	0
2878	29	78	1	0
2879	29	79	0	0
2880	29	80	1	0
2881	29	81	0	0
2882	29	82	1	0
2883	29	83	1	0

Observation	Subject	Trial	Correct	Pressure
2884	29	84	1	0
2885	29	85	0	0
2886	29	86	1	0
2887	29	87	0	0
2888	29	88	0	0
2889	29	89	0	0
2890	29	90	0	0
2891	29	91	0	0
2892	29	92	0	0
2893	29	93	1	0
2894	29	94	0	0
2895	29	95	1	0
2896	29	96	1	0
2897	29	97	0	0
2898	29	98	0	0
2899	29	99	0	0
2900	29	100	0	0
2901	30	1	1	-25
2902	30	2	1	25
2903	30	3	1	-25
2904	30	4	1	-25
2905	30	5	1	25
2906	30	6	1	-25
2907	30	7	1	25
2908	30	8	0	-25
2909	30	9	1	25
2910	30	10	1	25
2911	30	11	1	-25
2912	30	12	0	25
2913	30	13	1	-25
2914	30	14	1	-25
2915	30	15	1	25
2916	30	16	0	-25
2917	30	17	1	25
2918	30	18	1	25
2919	30	19	0	-25
2920	30	20	1	-25
2921	30	21	1	25
2922	30	22	1	-25
2923	30	23	0	25
2924	30	24	1	-25
2925	30	25	1	25
2926	30	26	0	25
2927	30	27	1	-25

Observation	Subject	Trial	Correct	Pressure
2928	30	28	1	25
2929	30	29	0	-25
2930	30	30	1	25
2931	30	31	1	-25
2932	30	32	1	25
2933	30	33	0	-25
2934	30	34	1	-25
2935	30	35	0	25
2936	30	36	1	25
2937	30	37	1	-25
2938	30	38	0	25
2939	30	39	0	-25
2940	30	40	1	-25
2941	30	41	0	25
2942	30	42	1	-25
2943	30	43	1	25
2944	30	44	1	-25
2945	30	45	1	25
2946	30	46	0	25
2947	30	47	1	-25
2948	30	48	1	25
2949	30	49	0	-25
2950	30	50	1	25
2951	30	51	0	0
2952	30	52	0	0
2953	30	53	1	0
2954	30	54	1	0
2955	30	55	0	0
2956	30	56	0	0
2957	30	57	1	0
2958	30	58	0	0
2959	30	59	0	0
2960	30	60	0	0
2961	30	61	1	0
2962	30	62	1	0
2963	30	63	1	0
2964	30	64	0	0
2965	30	65	1	0
2966	30	66	0	0
2967	30	67	1	0
2968	30	68	0	0
2969	30	69	1	0
2970	30	70	1	0
2971	30	71	1	0

Observation	Subject	Trial	Correct	Pressure
2972	30	72	0	0
2973	30	73	1	0
2974	30	74	1	0
2975	30	75	0	0
2976	30	76	0	0
2977	30	77	1	0
2978	30	78	1	0
2979	30	79	0	0
2980	30	80	1	0
2981	30	81	0	0
2982	30	82	1	0
2983	30	83	1	0
2984	30	84	1	0
2985	30	85	0	0
2986	30	86	1	0
2987	30	87	0	0
2988	30	88	0	0
2989	30	89	0	0
2990	30	90	0	0
2991	30	91	1	0
2992	30	92	0	0
2993	30	93	1	0
2994	30	94	0	0
2995	30	95	1	0
2996	30	96	1	0
2997	30	97	1	0
2998	30	98	0	0
2999	30	99	1	0
3000	30	100	0	0
3001	31	1	1	-25
3002	31	2	0	25
3003	31	3	1	-25
3004	31	4	1	-25
3005	31	5	0	25
3006	31	6	1	-25
3007	31	7	1	25
3008	31	8	1	-25
3009	31	9	1	25
3010	31	10	0	25
3011	31	11	0	-25
3012	31	12	0	25
3013	31	13	0	-25
3014	31	14	0	-25
3015	31	15	1	25

Observation	Subject	Trial	Correct	Pressure
3016	31	16	0	-25
3017	31	17	0	25
3018	31	18	1	25
3019	31	19	1	-25
3020	31	20	0	-25
3021	31	21	0	25
3022	31	22	0	-25
3023	31	23	1	25
3024	31	24	1	-25
3025	31	25	0	25
3026	31	26	0	25
3027	31	27	0	-25
3028	31	28	1	25
3029	31	29	0	-25
3030	31	30	1	25
3031	31	31	1	-25
3032	31	32	0	25
3033	31	33	0	-25
3034	31	34	1	-25
3035	31	35	1	25
3036	31	36	1	25
3037	31	37	1	-25
3038	31	38	0	25
3039	31	39	1	-25
3040	31	40	1	-25
3041	31	41	0	25
3042	31	42	1	-25
3043	31	43	1	25
3044	31	44	0	-25
3045	31	45	0	25
3046	31	46	0	25
3047	31	47	1	-25
3048	31	48	1	25
3049	31	49	1	-25
3050	31	50	0	25
3051	31	51	1	0
3052	31	52	0	0
3053	31	53	1	0
3054	31	54	1	0
3055	31	55	0	0
3056	31	56	0	0
3057	31	57	1	0
3058	31	58	0	0
3059	31	59	1	0

Observation	Subject	Trial	Correct	Pressure
3060	31	60	0	0
3061	31	61	1	0
3062	31	62	1	0
3063	31	63	1	0
3064	31	64	0	0
3065	31	65	1	0
3066	31	66	0	0
3067	31	67	1	0
3068	31	68	0	0
3069	31	69	1	0
3070	31	70	0	0
3071	31	71	1	0
3072	31	72	1	0
3073	31	73	1	0
3074	31	74	0	0
3075	31	75	1	0
3076	31	76	0	0
3077	31	77	1	0
3078	31	78	1	0
3079	31	79	0	0
3080	31	80	1	0
3081	31	81	0	0
3082	31	82	1	0
3083	31	83	1	0
3084	31	84	1	0
3085	31	85	1	0
3086	31	86	1	0
3087	31	87	1	0
3088	31	88	1	0
3089	31	89	0	0
3090	31	90	0	0
3091	31	91	1	0
3092	31	92	1	0
3093	31	93	1	0
3094	31	94	0	0
3095	31	95	1	0
3096	31	96	1	0
3097	31	97	0	0
3098	31	98	0	0
3099	31	99	0	0
3100	31	100	0	0
3101	32	1	1	-25
3102	32	2	0	25
3103	32	3	1	-25

Observation	Subject	Trial	Correct	Pressure
3104	32	4	1	-25
3105	32	5	1	25
3106	32	6	1	-25
3107	32	7	1	25
3108	32	8	0	-25
3109	32	9	1	25
3110	32	10	1	25
3111	32	11	1	-25
3112	32	12	1	25
3113	32	13	0	-25
3114	32	14	0	-25
3115	32	15	1	25
3116	32	16	0	-25
3117	32	17	1	25
3118	32	18	1	25
3119	32	19	1	-25
3120	32	20	1	-25
3121	32	21	1	25
3122	32	22	1	-25
3123	32	23	1	25
3124	32	24	1	-25
3125	32	25	0	25
3126	32	26	1	25
3127	32	27	1	-25
3128	32	28	1	25
3129	32	29	1	-25
3130	32	30	1	25
3131	32	31	1	-25
3132	32	32	1	25
3133	32	33	0	-25
3134	32	34	1	-25
3135	32	35	1	25
3136	32	36	1	25
3137	32	37	1	-25
3138	32	38	1	25
3139	32	39	1	-25
3140	32	40	1	-25
3141	32	41	1	25
3142	32	42	0	-25
3143	32	43	0	25
3144	32	44	1	-25
3145	32	45	1	25
3146	32	46	0	25
3147	32	47	1	-25

Observation	Subject	Trial	Correct	Pressure
3148	32	48	1	25
3149	32	49	1	-25
3150	32	50	0	25
3151	32	51	1	0
3152	32	52	0	0
3153	32	53	1	0
3154	32	54	1	0
3155	32	55	1	0
3156	32	56	0	0
3157	32	57	1	0
3158	32	58	1	0
3159	32	59	0	0
3160	32	60	0	0
3161	32	61	1	0
3162	32	62	1	0
3163	32	63	1	0
3164	32	64	0	0
3165	32	65	1	0
3166	32	66	0	0
3167	32	67	1	0
3168	32	68	1	0
3169	32	69	1	0
3170	32	70	1	0
3171	32	71	1	0
3172	32	72	1	0
3173	32	73	0	0
3174	32	74	1	0
3175	32	75	1	0
3176	32	76	0	0
3177	32	77	1	0
3178	32	78	0	0
3179	32	79	0	0
3180	32	80	1	0
3181	32	81	0	0
3182	32	82	1	0
3183	32	83	1	0
3184	32	84	1	0
3185	32	85	1	0
3186	32	86	1	0
3187	32	87	0	0
3188	32	88	0	0
3189	32	89	1	0
3190	32	90	1	0
3191	32	91	0	0

Observation	Subject	Trial	Correct	Pressure
3192	32	92	0	0
3193	32	93	1	0
3194	32	94	1	0
3195	32	95	1	0
3196	32	96	1	0
3197	32	97	0	0
3198	32	98	0	0
3199	32	99	1	0
3200	32	100	1	0
3201	33	1	1	0
3202	33	2	0	0
3203	33	3	1	0
3204	33	4	1	0
3205	33	5	1	0
3206	33	6	1	0
3207	33	7	1	0
3208	33	8	1	0
3209	33	9	1	0
3210	33	10	1	0
3211	33	11	1	0
3212	33	12	0	0
3213	33	13	1	0
3214	33	14	0	0
3215	33	15	1	0
3216	33	16	1	0
3217	33	17	1	0
3218	33	18	1	0
3219	33	19	1	0
3220	33	20	0	0
3221	33	21	1	0
3222	33	22	0	0
3223	33	23	1	0
3224	33	24	0	0
3225	33	25	0	0
3226	33	26	1	0
3227	33	27	0	0
3228	33	28	1	0
3229	33	29	0	0
3230	33	30	0	0
3231	33	31	1	0
3232	33	32	0	0
3233	33	33	0	0
3234	33	34	1	0
3235	33	35	0	0

Observation	Subject	Trial	Correct	Pressure
3236	33	36	0	0
3237	33	37	1	0
3238	33	38	1	0
3239	33	39	0	0
3240	33	40	1	0
3241	33	41	0	0
3242	33	42	1	0
3243	33	43	1	0
3244	33	44	0	0
3245	33	45	1	0
3246	33	46	0	0
3247	33	47	1	0
3248	33	48	1	0
3249	33	49	1	0
3250	33	50	1	0
3251	33	51	1	-25
3252	33	52	1	25
3253	33	53	1	-25
3254	33	54	1	-25
3255	33	55	1	25
3256	33	56	1	-25
3257	33	57	1	25
3258	33	58	0	-25
3259	33	59	0	25
3260	33	60	0	25
3261	33	61	1	-25
3262	33	62	1	25
3263	33	63	1	-25
3264	33	64	0	-25
3265	33	65	0	25
3266	33	67	1	25
3267	33	68	1	25
3268	33	69	1	-25
3269	33	70	1	-25
3270	33	71	1	25
3271	33	72	0	-25
3272	33	73	1	25
3273	33	74	0	-25
3274	33	75	0	25
3275	33	76	0	25
3276	33	77	1	-25
3277	33	78	1	25
3278	33	79	0	-25
3279	33	80	1	25

Observation	Subject	Trial	Correct	Pressure
3280	33	81	0	-25
3281	33	82	1	25
3282	33	83	1	-25
3283	33	84	0	-25
3284	33	85	0	25
3285	33	86	1	25
3286	33	87	1	-25
3287	33	88	0	25
3288	33	89	0	-25
3289	33	90	0	-25
3290	33	91	0	25
3291	33	92	0	-25
3292	33	93	1	25
3293	33	94	0	-25
3294	33	95	1	25
3295	33	96	0	25
3296	33	97	1	-25
3297	33	98	0	25
3298	33	99	1	-25
3299	33	100	1	25
3300	34	1	1	0
3301	34	2	0	0
3302	34	3	1	0
3303	34	4	1	0
3304	34	5	0	0
3305	34	6	1	0
3306	34	7	1	0
3307	34	8	0	0
3308	34	9	1	0
3309	34	10	1	0
3310	34	11	1	0
3311	34	12	0	0
3312	34	13	1	0
3313	34	14	0	0
3314	34	15	1	0
3315	34	16	0	0
3316	34	17	1	0
3317	34	18	1	0
3318	34	19	0	0
3319	34	20	0	0
3320	34	21	1	0
3321	34	22	0	0
3322	34	23	1	0
3323	34	24	1	0

Observation	Subject	Trial	Correct	Pressure
3324	34	25	0	0
3325	34	26	0	0
3326	34	27	1	0
3327	34	28	1	0
3328	34	29	0	0
3329	34	30	0	0
3330	34	31	1	0
3331	34	32	0	0
3332	34	33	0	0
3333	34	34	1	0
3334	34	35	0	0
3335	34	36	0	0
3336	34	37	1	0
3337	34	38	0	0
3338	34	39	1	0
3339	34	40	1	0
3340	34	41	1	0
3341	34	42	0	0
3342	34	43	1	0
3343	34	44	0	0
3344	34	45	1	0
3345	34	46	1	0
3346	34	47	1	0
3347	34	48	1	0
3348	34	49	1	0
3349	34	50	0	0
3350	34	51	0	-25
3351	34	52	0	25
3352	34	53	1	-25
3353	34	54	1	-25
3354	34	55	0	25
3355	34	56	1	-25
3356	34	57	1	25
3357	34	58	1	-25
3358	34	59	0	25
3359	34	60	0	25
3360	34	61	1	-25
3361	34	62	1	25
3362	34	63	1	-25
3363	34	64	1	-25
3364	34	65	0	25
3365	34	66	0	-25
3366	34	67	1	25
3367	34	68	1	25

Observation	Subject	Trial	Correct	Pressure
3368	34	69	1	-25
3369	34	70	1	-25
3370	34	71	1	25
3371	34	72	0	-25
3372	34	73	1	25
3373	34	74	0	-25
3374	34	75	1	25
3375	34	76	0	25
3376	34	77	1	-25
3377	34	78	0	25
3378	34	79	0	-25
3379	34	80	1	25
3380	34	81	0	-25
3381	34	82	1	25
3382	34	83	1	-25
3383	34	84	1	-25
3384	34	85	0	25
3385	34	86	1	25
3386	34	87	0	-25
3387	34	88	1	25
3388	34	89	1	-25
3389	34	90	0	-25
3390	34	91	0	25
3391	34	92	0	-25
3392	34	93	1	25
3393	34	94	1	-25
3394	34	95	1	25
3395	34	96	1	25
3396	34	97	0	-25
3397	34	98	1	25
3398	34	99	1	-25
3399	34	100	1	25
3400	35	1	1	0
3401	35	2	0	0
3402	35	3	1	0
3403	35	4	1	0
3404	35	5	0	0
3405	35	6	1	0
3406	35	7	1	0
3407	35	8	0	0
3408	35	9	0	0
3409	35	10	1	0
3410	35	11	1	0
3411	35	12	0	0

Observation	Subject	Trial	Correct	Pressure
3412	35	13	0	0
3413	35	14	0	0
3414	35	15	1	0
3415	35	16	1	0
3416	35	17	1	0
3417	35	18	1	0
3418	35	19	1	0
3419	35	20	0	0
3420	35	21	1	0
3421	35	22	1	0
3422	35	23	0	0
3423	35	24	1	0
3424	35	25	0	0
3425	35	26	0	0
3426	35	27	0	0
3427	35	28	1	0
3428	35	29	0	0
3429	35	30	0	0
3430	35	31	1	0
3431	35	32	0	0
3432	35	33	0	0
3433	35	34	1	0
3434	35	35	1	0
3435	35	36	0	0
3436	35	37	1	0
3437	35	38	0	0
3438	35	39	1	0
3439	35	40	1	0
3440	35	41	0	0
3441	35	42	0	0
3442	35	43	1	0
3443	35	44	0	0
3444	35	45	1	0
3445	35	46	1	0
3446	35	47	0	0
3447	35	48	1	0
3448	35	49	1	0
3449	35	50	1	0
3450	35	51	0	-25
3451	35	52	0	25
3452	35	53	1	-25
3453	35	54	0	-25
3454	35	55	0	25
3455	35	56	0	-25

Observation	Subject	Trial	Correct	Pressure
3456	35	57	1	25
3457	35	58	0	-25
3458	35	59	0	25
3459	35	60	0	25
3460	35	61	1	-25
3461	35	62	1	25
3462	35	63	1	-25
3463	35	64	0	-25
3464	35	65	0	25
3465	35	66	0	-25
3466	35	67	0	25
3467	35	68	1	25
3468	35	69	1	-25
3469	35	70	0	-25
3470	35	71	0	25
3471	35	72	0	-25
3472	35	73	1	25
3473	35	74	0	-25
3474	35	75	1	25
3475	35	76	0	25
3476	35	77	1	-25
3477	35	78	1	25
3478	35	79	0	-25
3479	35	80	1	25
3480	35	81	0	-25
3481	35	82	1	25
3482	35	83	1	-25
3483	35	84	1	-25
3484	35	85	0	25
3485	35	86	1	25
3486	35	87	0	-25
3487	35	88	0	25
3488	35	89	0	-25
3489	35	90	0	-25
3490	35	91	0	25
3491	35	92	0	-25
3492	35	93	1	25
3493	35	94	0	-25
3494	35	95	1	25
3495	35	96	1	25
3496	35	97	1	-25
3497	35	98	0	25
3498	35	99	0	-25
3499	35	100	0	25

Observation	Subject	Trial	Correct	Pressure
3500	36	1	1	0
3501	36	2	0	0
3502	36	3	0	0
3503	36	4	1	0
3504	36	5	0	0
3505	36	6	0	0
3506	36	7	1	0
3507	36	8	0	0
3508	36	9	1	0
3509	36	10	1	0
3510	36	11	1	0
3511	36	12	1	0
3512	36	13	0	0
3513	36	14	0	0
3514	36	15	1	0
3515	36	16	1	0
3516	36	17	0	0
3517	36	18	1	0
3518	36	19	0	0
3519	36	20	0	0
3520	36	21	1	0
3521	36	22	1	0
3522	36	23	0	0
3523	36	24	0	0
3524	36	25	0	0
3525	36	26	0	0
3526	36	27	0	0
3527	36	28	1	0
3528	36	29	0	0
3529	36	30	0	0
3530	36	31	1	0
3531	36	32	0	0
3532	36	33	0	0
3533	36	34	1	0
3534	36	35	1	0
3535	36	36	1	0
3536	36	37	1	0
3537	36	38	1	0
3538	36	39	1	0
3539	36	40	1	0
3540	36	41	0	0
3541	36	42	1	0
3542	36	43	1	0
3543	36	44	1	0

Observation	Subject	Trial	Correct	Pressure
3544	36	45	1	0
3545	36	46	0	0
3546	36	47	1	0
3547	36	48	1	0
3548	36	49	1	0
3549	36	50	1	0
3550	36	51	0	-25
3551	36	52	0	25
3552	36	53	1	-25
3553	36	54	1	-25
3554	36	55	0	25
3555	36	56	1	-25
3556	36	57	1	25
3557	36	58	0	-25
3558	36	59	0	25
3559	36	60	0	25
3560	36	61	1	-25
3561	36	62	1	25
3562	36	63	1	-25
3563	36	64	0	-25
3564	36	65	0	25
3565	36	66	0	-25
3566	36	67	1	25
3567	36	68	0	25
3568	36	69	1	-25
3569	36	70	0	-25
3570	36	71	1	25
3571	36	72	0	-25
3572	36	73	0	25
3573	36	74	1	-25
3574	36	75	1	25
3575	36	76	0	25
3576	36	77	1	-25
3577	36	78	1	25
3578	36	79	0	-25
3579	36	80	1	25
3580	36	81	1	-25
3581	36	82	1	25
3582	36	83	1	-25
3583	36	84	1	-25
3584	36	85	0	25
3585	36	86	1	25
3586	36	87	1	-25
3587	36	88	1	25

Observation	Subject	Trial	Correct	Pressure
3588	36	89	1	-25
3589	36	90	0	-25
3590	36	91	1	25
3591	36	92	0	-25
3592	36	93	1	25
3593	36	94	0	-25
3594	36	95	1	25
3595	36	96	0	25
3596	36	97	0	-25
3597	36	98	0	25
3598	36	99	1	-25
3599	36	100	1	25
3600	37	1	1	-25
3601	37	2	0	25
3602	37	3	1	-25
3603	37	4	1	-25
3604	37	5	1	25
3605	37	6	0	-25
3606	37	7	1	25
3607	37	8	0	-25
3608	37	9	0	25
3609	37	10	1	25
3610	37	11	1	-25
3611	37	12	1	25
3612	37	13	1	-25
3613	37	14	0	-25
3614	37	15	1	25
3615	37	16	0	-25
3616	37	17	0	25
3617	37	18	1	25
3618	37	19	1	-25
3619	37	20	0	-25
3620	37	21	1	25
3621	37	22	1	-25
3622	37	23	1	25
3623	37	24	1	-25
3624	37	25	1	25
3625	37	26	0	25
3626	37	27	0	-25
3627	37	28	1	25
3628	37	29	0	-25
3629	37	30	0	25
3630	37	31	1	-25
3631	37	32	0	25

Observation	Subject	Trial	Correct	Pressure
3632	37	33	0	-25
3633	37	34	1	-25
3634	37	35	0	25
3635	37	36	1	25
3636	37	37	1	-25
3637	37	38	0	25
3638	37	39	1	-25
3639	37	40	0	-25
3640	37	41	0	25
3641	37	42	1	-25
3642	37	43	0	25
3643	37	44	0	-25
3644	37	45	1	25
3645	37	46	1	25
3646	37	47	1	-25
3647	37	48	1	25
3648	37	49	1	-25
3649	37	50	0	25
3650	37	51	1	0
3651	37	52	1	0
3652	37	53	1	0
3653	37	54	1	0
3654	37	55	0	0
3655	37	56	0	0
3656	37	57	1	0
3657	37	58	0	0
3658	37	59	0	0
3659	37	60	0	0
3660	37	61	1	0
3661	37	62	1	0
3662	37	63	1	0
3663	37	64	0	0
3664	37	65	1	0
3665	37	66	0	0
3666	37	67	1	0
3667	37	68	0	0
3668	37	69	1	0
3669	37	70	1	0
3670	37	71	1	0
3671	37	72	0	0
3672	37	73	1	0
3673	37	74	1	0
3674	37	75	0	0
3675	37	76	0	0

Observation	Subject	Trial	Correct	Pressure
3676	37	77	1	0
3677	37	78	0	0
3678	37	79	0	0
3679	37	80	0	0
3680	37	81	0	0
3681	37	82	1	0
3682	37	83	1	0
3683	37	84	1	0
3684	37	85	0	0
3685	37	86	1	0
3686	37	87	0	0
3687	37	88	0	0
3688	37	89	1	0
3689	37	90	0	0
3690	37	91	0	0
3691	37	92	0	0
3692	37	93	1	0
3693	37	94	0	0
3694	37	95	1	0
3695	37	96	1	0
3696	37	97	0	0
3697	37	98	0	0
3698	37	99	0	0
3699	37	100	1	0
3700	38	1	1	-25
3701	38	2	0	25
3702	38	3	1	-25
3703	38	4	1	-25
3704	38	5	0	25
3705	38	6	1	-25
3706	38	7	1	25
3707	38	8	0	-25
3708	38	9	1	25
3709	38	10	0	25
3710	38	11	1	-25
3711	38	12	0	25
3712	38	13	0	-25
3713	38	14	0	-25
3714	38	15	1	25
3715	38	16	1	-25
3716	38	17	0	25
3717	38	18	1	25
3718	38	19	1	-25
3719	38	20	0	-25

Observation	Subject	Trial	Correct	Pressure
3720	38	21	1	25
3721	38	22	1	-25
3722	38	23	1	25
3723	38	24	1	-25
3724	38	25	1	25
3725	38	26	1	25
3726	38	27	1	-25
3727	38	28	1	25
3728	38	29	0	-25
3729	38	30	0	25
3730	38	31	1	-25
3731	38	32	1	25
3732	38	33	1	-25
3733	38	34	1	-25
3734	38	35	0	25
3735	38	36	1	25
3736	38	37	1	-25
3737	38	38	0	25
3738	38	39	1	-25
3739	38	40	1	-25
3740	38	41	0	25
3741	38	42	0	-25
3742	38	43	1	25
3743	38	44	0	-25
3744	38	45	1	25
3745	38	46	1	25
3746	38	47	1	-25
3747	38	48	1	25
3748	38	49	1	-25
3749	38	50	1	25
3750	38	51	0	0
3751	38	52	0	0
3752	38	53	0	0
3753	38	54	1	0
3754	38	55	0	0
3755	38	56	0	0
3756	38	57	1	0
3757	38	58	0	0
3758	38	59	0	0
3759	38	60	0	0
3760	38	61	1	0
3761	38	62	1	0
3762	38	63	1	0
3763	38	64	0	0

Observation	Subject	Trial	Correct	Pressure
3764	38	65	1	0
3765	38	66	1	0
3766	38	67	1	0
3767	38	68	0	0
3768	38	69	1	0
3769	38	70	1	0
3770	38	71	1	0
3771	38	72	1	0
3772	38	73	1	0
3773	38	74	0	0
3774	38	75	0	0
3775	38	76	0	0
3776	38	77	1	0
3777	38	78	1	0
3778	38	79	0	0
3779	38	80	1	0
3780	38	81	0	0
3781	38	82	1	0
3782	38	83	1	0
3783	38	84	1	0
3784	38	85	1	0
3785	38	86	1	0
3786	38	87	0	0
3787	38	88	0	0
3788	38	89	0	0
3789	38	90	1	0
3790	38	91	1	0
3791	38	92	0	0
3792	38	93	1	0
3793	38	94	0	0
3794	38	95	1	0
3795	38	96	1	0
3796	38	97	0	0
3797	38	98	0	0
3798	38	99	1	0
3799	38	100	1	0
3800	39	1	1	-25
3801	39	2	0	25
3802	39	3	1	-25
3803	39	4	1	-25
3804	39	5	0	25
3805	39	6	1	-25
3806	39	7	1	25
3807	39	8	1	-25

Observation	Subject	Trial	Correct	Pressure
3808	39	9	1	25
3809	39	10	1	25
3810	39	11	0	-25
3811	39	12	0	25
3812	39	13	1	-25
3813	39	14	0	-25
3814	39	15	1	25
3815	39	16	0	-25
3816	39	17	1	25
3817	39	18	0	25
3818	39	19	1	-25
3819	39	20	0	-25
3820	39	21	1	25
3821	39	22	1	-25
3822	39	23	1	25
3823	39	24	1	-25
3824	39	25	1	25
3825	39	26	0	25
3826	39	27	1	-25
3827	39	28	1	25
3828	39	29	0	-25
3829	39	30	0	25
3830	39	31	1	-25
3831	39	32	0	25
3832	39	33	0	-25
3833	39	34	1	-25
3834	39	35	1	25
3835	39	36	1	25
3836	39	37	1	-25
3837	39	38	0	25
3838	39	39	1	-25
3839	39	40	1	-25
3840	39	41	0	25
3841	39	42	1	-25
3842	39	43	1	25
3843	39	44	0	-25
3844	39	45	1	25
3845	39	46	0	25
3846	39	47	1	-25
3847	39	48	1	25
3848	39	49	1	-25
3849	39	50	0	25
3850	39	51	0	0
3851	39	52	1	0

Observation	Subject	Trial	Correct	Pressure
3852	39	53	1	0
3853	39	54	1	0
3854	39	55	0	0
3855	39	56	0	0
3856	39	57	1	0
3857	39	58	1	0
3858	39	59	1	0
3859	39	60	1	0
3860	39	61	1	0
3861	39	62	1	0
3862	39	63	1	0
3863	39	64	1	0
3864	39	65	0	0
3865	39	66	1	0
3866	39	67	1	0
3867	39	68	0	0
3868	39	69	1	0
3869	39	70	0	0
3870	39	71	1	0
3871	39	72	1	0
3872	39	73	0	0
3873	39	74	0	0
3874	39	75	0	0
3875	39	76	1	0
3876	39	77	1	0
3877	39	78	1	0
3878	39	79	0	0
3879	39	80	1	0
3880	39	81	0	0
3881	39	82	1	0
3882	39	83	1	0
3883	39	84	1	0
3884	39	85	1	0
3885	39	86	1	0
3886	39	87	0	0
3887	39	88	1	0
3888	39	89	0	0
3889	39	90	0	0
3890	39	91	0	0
3891	39	92	1	0
3892	39	93	1	0
3893	39	94	0	0
3894	39	95	1	0
3895	39	96	1	0

Observation	Subject	Trial	Correct	Pressure
3896	39	97	0	0
3897	39	98	0	0
3898	39	99	1	0
3899	39	100	1	0
3900	40	1	1	-25
3901	40	2	0	25
3902	40	3	1	-25
3903	40	4	1	-25
3904	40	5	1	25
3905	40	6	0	-25
3906	40	7	1	25
3907	40	8	0	-25
3908	40	9	0	25
3909	40	10	1	25
3910	40	11	0	-25
3911	40	12	0	25
3912	40	13	0	-25
3913	40	14	0	-25
3914	40	15	1	25
3915	40	16	0	-25
3916	40	17	1	25
3917	40	18	1	25
3918	40	19	1	-25
3919	40	20	0	-25
3920	40	21	0	25
3921	40	22	0	-25
3922	40	23	0	25
3923	40	24	1	-25
3924	40	25	0	25
3925	40	26	0	25
3926	40	27	0	-25
3927	40	28	1	25
3928	40	29	0	-25
3929	40	30	0	25
3930	40	31	1	-25
3931	40	32	1	25
3932	40	33	0	-25
3933	40	34	1	-25
3934	40	35	0	25
3935	40	36	1	25
3936	40	37	1	-25
3937	40	38	0	25
3938	40	39	0	-25
3939	40	40	0	-25

Observation	Subject	Trial	Correct	Pressure
3940	40	41	0	25
3941	40	42	0	-25
3942	40	43	0	25
3943	40	44	1	-25
3944	40	45	1	25
3945	40	46	1	25
3946	40	47	1	-25
3947	40	48	1	25
3948	40	49	0	-25
3949	40	50	0	25
3950	40	51	1	0
3951	40	52	0	0
3952	40	53	1	0
3953	40	54	1	0
3954	40	55	0	0
3955	40	56	0	0
3956	40	57	1	0
3957	40	58	1	0
3958	40	59	0	0
3959	40	60	0	0
3960	40	61	1	0
3961	40	62	1	0
3962	40	63	1	0
3963	40	64	1	0
3964	40	65	0	0
3965	40	66	1	0
3966	40	67	0	0
3967	40	68	0	0
3968	40	69	1	0
3969	40	70	1	0
3970	40	71	1	0
3971	40	72	0	0
3972	40	73	0	0
3973	40	74	0	0
3974	40	75	0	0
3975	40	76	0	0
3976	40	77	1	0
3977	40	78	1	0
3978	40	79	0	0
3979	40	80	1	0
3980	40	81	0	0
3981	40	82	1	0
3982	40	83	1	0
3983	40	84	1	0

Observation	Subject	Trial	Correct	Pressure
3984	40	85	0	0
3985	40	86	1	0
3986	40	87	0	0
3987	40	88	0	0
3988	40	89	0	0
3989	40	90	0	0
3990	40	91	0	0
3991	40	92	0	0
3992	40	93	1	0
3993	40	94	0	0
3994	40	95	1	0
3995	40	96	1	0
3996	40	97	0	0
3997	40	98	1	0
3998	40	99	0	0
3999	40	100	0	0
4000	41	1	1	0
4001	41	2	0	0
4002	41	3	0	0
4003	41	4	1	0
4004	41	5	0	0
4005	41	6	0	0
4006	41	7	1	0
4007	41	8	1	0
4008	41	9	0	0
4009	41	10	1	0
4010	41	11	1	0
4011	41	12	1	0
4012	41	13	1	0
4013	41	14	1	0
4014	41	15	1	0
4015	41	16	1	0
4016	41	17	0	0
4017	41	18	1	0
4018	41	19	0	0
4019	41	20	1	0
4020	41	21	0	0
4021	41	22	0	0
4022	41	23	1	0
4023	41	24	1	0
4024	41	25	0	0
4025	41	26	1	0
4026	41	27	1	0
4027	41	28	1	0

Observation	Subject	Trial	Correct	Pressure
4028	41	29	0	0
4029	41	30	0	0
4030	41	31	1	0
4031	41	32	0	0
4032	41	33	0	0
4033	41	34	1	0
4034	41	35	1	0
4035	41	36	0	0
4036	41	37	1	0
4037	41	38	0	0
4038	41	39	0	0
4039	41	40	0	0
4040	41	41	1	0
4041	41	42	1	0
4042	41	43	1	0
4043	41	44	0	0
4044	41	45	1	0
4045	41	46	1	0
4046	41	47	1	0
4047	41	48	1	0
4048	41	49	1	0
4049	41	50	1	0
4050	41	51	1	-25
4051	41	52	0	25
4052	41	53	1	-25
4053	41	54	1	-25
4054	41	55	0	25
4055	41	56	0	-25
4056	41	57	1	25
4057	41	58	1	-25
4058	41	59	0	25
4059	41	60	0	25
4060	41	61	1	-25
4061	41	62	1	25
4062	41	63	1	-25
4063	41	64	1	-25
4064	41	65	0	25
4065	41	66	0	-25
4066	41	67	1	25
4067	41	68	1	25
4068	41	69	1	-25
4069	41	70	0	-25
4070	41	71	1	25
4071	41	72	1	-25

Observation	Subject	Trial	Correct	Pressure
4072	41	73	0	25
4073	41	74	0	-25
4074	41	75	0	25
4075	41	76	0	25
4076	41	77	1	-25
4077	41	78	1	25
4078	41	79	0	-25
4079	41	80	1	25
4080	41	81	0	-25
4081	41	82	1	25
4082	41	83	1	-25
4083	41	84	1	-25
4084	41	85	0	25
4085	41	86	1	25
4086	41	87	0	-25
4087	41	88	0	25
4088	41	89	1	-25
4089	41	90	0	-25
4090	41	91	0	25
4091	41	92	0	-25
4092	41	93	1	25
4093	41	94	0	-25
4094	41	95	1	25
4095	41	96	0	25
4096	41	97	0	-25
4097	41	98	0	25
4098	41	99	0	-25
4099	41	100	1	25
4100	42	1	1	0
4101	42	2	1	0
4102	42	3	1	0
4103	42	4	1	0
4104	42	5	0	0
4105	42	6	1	0
4106	42	7	1	0
4107	42	8	0	0
4108	42	9	1	0
4109	42	10	1	0
4110	42	11	1	0
4111	42	12	1	0
4112	42	13	1	0
4113	42	14	0	0
4114	42	15	1	0
4115	42	16	1	0

Observation	Subject	Trial	Correct	Pressure
4116	42	17	1	0
4117	42	18	1	0
4118	42	19	1	0
4119	42	20	1	0
4120	42	21	1	0
4121	42	22	1	0
4122	42	23	0	0
4123	42	24	1	0
4124	42	25	0	0
4125	42	26	0	0
4126	42	27	0	0
4127	42	28	1	0
4128	42	29	0	0
4129	42	30	0	0
4130	42	31	1	0
4131	42	32	1	0
4132	42	34	1	0
4133	42	35	1	0
4134	42	36	0	0
4135	42	37	1	0
4136	42	38	0	0
4137	42	39	1	0
4138	42	40	0	0
4139	42	41	0	0
4140	42	42	1	0
4141	42	43	0	0
4142	42	44	1	0
4143	42	45	1	0
4144	42	46	1	0
4145	42	47	1	0
4146	42	48	1	0
4147	42	49	0	0
4148	42	50	0	0
4149	42	51	1	-25
4150	42	52	0	25
4151	42	53	1	-25
4152	42	54	1	-25
4153	42	55	0	25
4154	42	56	1	-25
4155	42	57	0	25
4156	42	58	0	-25
4157	42	59	1	25
4158	42	60	1	25
4159	42	61	1	-25

Observation	Subject	Trial	Correct	Pressure
4160	42	62	1	25
4161	42	63	1	-25
4162	42	64	1	-25
4163	42	65	1	25
4164	42	66	0	-25
4165	42	67	0	25
4166	42	68	0	25
4167	42	69	1	-25
4168	42	70	0	-25
4169	42	71	1	25
4170	42	72	0	-25
4171	42	73	1	25
4172	42	75	0	25
4173	42	76	1	25
4174	42	77	1	-25
4175	42	78	1	25
4176	42	79	0	-25
4177	42	80	1	25
4178	42	81	1	-25
4179	42	82	1	25
4180	42	83	1	-25
4181	42	84	1	-25
4182	42	85	0	25
4183	42	86	1	25
4184	42	87	0	-25
4185	42	88	1	25
4186	42	89	1	-25
4187	42	90	0	-25
4188	42	91	0	25
4189	42	92	0	-25
4190	42	93	1	25
4191	42	94	1	-25
4192	42	95	1	25
4193	42	96	1	25
4194	42	97	0	-25
4195	42	98	0	25
4196	42	99	0	-25
4197	42	100	0	25
4198	43	1	1	0
4199	43	2	0	0
4200	43	3	1	0
4201	43	4	1	0
4202	43	5	0	0
4203	43	6	1	0

Observation	Subject	Trial	Correct	Pressure
4204	43	7	1	0
4205	43	8	0	0
4206	43	9	1	0
4207	43	10	1	0
4208	43	11	1	0
4209	43	12	1	0
4210	43	13	0	0
4211	43	14	0	0
4212	43	15	1	0
4213	43	16	1	0
4214	43	17	1	0
4215	43	18	1	0
4216	43	19	1	0
4217	43	20	0	0
4218	43	21	0	0
4219	43	22	1	0
4220	43	23	1	0
4221	43	24	1	0
4222	43	25	0	0
4223	43	26	1	0
4224	43	27	0	0
4225	43	28	1	0
4226	43	29	0	0
4227	43	30	0	0
4228	43	31	1	0
4229	43	32	0	0
4230	43	33	0	0
4231	43	34	1	0
4232	43	35	1	0
4233	43	36	1	0
4234	43	37	1	0
4235	43	38	0	0
4236	43	39	1	0
4237	43	40	0	0
4238	43	41	0	0
4239	43	42	1	0
4240	43	43	1	0
4241	43	44	1	0
4242	43	45	1	0
4243	43	46	0	0
4244	43	47	1	0
4245	43	48	1	0
4246	43	49	1	0
4247	43	50	1	0

Observation	Subject	Trial	Correct	Pressure
4248	43	51	1	-25
4249	43	52	0	25
4250	43	53	0	-25
4251	43	54	1	-25
4252	43	55	0	25
4253	43	56	0	-25
4254	43	57	0	25
4255	43	58	1	-25
4256	43	59	1	25
4257	43	60	1	25
4258	43	61	1	-25
4259	43	62	1	25
4260	43	63	1	-25
4261	43	64	0	-25
4262	43	65	1	25
4263	43	66	0	-25
4264	43	67	0	25
4265	43	68	0	25
4266	43	69	1	-25
4267	43	70	1	-25
4268	43	71	1	25
4269	43	72	0	-25
4270	43	73	1	25
4271	43	74	0	-25
4272	43	75	0	25
4273	43	76	1	25
4274	43	77	1	-25
4275	43	78	1	25
4276	43	79	0	-25
4277	43	80	1	25
4278	43	81	0	-25
4279	43	82	1	25
4280	43	83	1	-25
4281	43	84	1	-25
4282	43	85	1	25
4283	43	86	1	25
4284	43	87	0	-25
4285	43	88	0	25
4286	43	89	0	-25
4287	43	90	0	-25
4288	43	91	0	25
4289	43	92	0	-25
4290	43	93	0	25
4291	43	94	0	-25

Observation	Subject	Trial	Correct	Pressure
4292	43	95	1	25
4293	43	96	1	25
4294	43	97	0	-25
4295	43	98	0	25
4296	43	99	1	-25
4297	43	100	0	25
4298	44	1	1	0
4299	44	2	1	0
4300	44	3	0	0
4301	44	4	1	0
4302	44	5	0	0
4303	44	6	1	0
4304	44	7	0	0
4305	44	8	1	0
4306	44	9	1	0
4307	44	10	0	0
4308	44	11	1	0
4309	44	12	1	0
4310	44	13	0	0
4311	44	14	1	0
4312	44	15	1	0
4313	44	16	1	0
4314	44	17	1	0
4315	44	18	1	0
4316	44	19	1	0
4317	44	20	0	0
4318	44	21	0	0
4319	44	22	0	0
4320	44	23	1	0
4321	44	24	1	0
4322	44	25	0	0
4323	44	26	0	0
4324	44	27	1	0
4325	44	28	1	0
4326	44	29	0	0
4327	44	30	0	0
4328	44	31	1	0
4329	44	32	0	0
4330	44	33	0	0
4331	44	34	1	0
4332	44	35	1	0
4333	44	36	1	0
4334	44	37	1	0
4335	44	38	0	0

Observation	Subject	Trial	Correct	Pressure
4336	44	39	1	0
4337	44	40	1	0
4338	44	41	0	0
4339	44	42	0	0
4340	44	43	1	0
4341	44	44	0	0
4342	44	45	1	0
4343	44	46	1	0
4344	44	47	1	0
4345	44	48	1	0
4346	44	49	1	0
4347	44	50	1	0
4348	44	51	1	-25
4349	44	52	0	25
4350	44	53	0	-25
4351	44	54	1	-25
4352	44	55	1	25
4353	44	56	0	-25
4354	44	57	1	25
4355	44	58	0	-25
4356	44	59	0	25
4357	44	60	0	25
4358	44	61	1	-25
4359	44	62	1	25
4360	44	63	1	-25
4361	44	64	0	-25
4362	44	65	0	25
4363	44	66	0	-25
4364	44	67	0	25
4365	44	68	0	25
4366	44	69	1	-25
4367	44	70	0	-25
4368	44	71	1	25
4369	44	72	0	-25
4370	44	73	1	25
4371	44	74	0	-25
4372	44	75	0	25
4373	44	76	1	25
4374	44	77	1	-25
4375	44	78	1	25
4376	44	79	0	-25
4377	44	80	1	25
4378	44	81	0	-25
4379	44	82	1	25

Observation	Subject	Trial	Correct	Pressure
4380	44	83	1	-25
4381	44	84	1	-25
4382	44	85	0	25
4383	44	86	1	25
4384	44	87	0	-25
4385	44	88	1	25
4386	44	89	1	-25
4387	44	90	1	-25
4388	44	91	1	25
4389	44	92	0	-25
4390	44	93	1	25
4391	44	94	0	-25
4392	44	95	0	25
4393	44	96	1	25
4394	44	97	0	-25
4395	44	98	0	25
4396	44	99	0	-25
4397	44	100	0	25
4398	45	1	0	-25
4399	45	2	1	25
4400	45	3	1	-25
4401	45	4	1	-25
4402	45	5	0	25
4403	45	6	1	-25
4404	45	7	1	25
4405	45	8	1	-25
4406	45	9	1	25
4407	45	10	1	25
4408	45	11	0	-25
4409	45	12	1	25
4410	45	13	1	-25
4411	45	14	1	-25
4412	45	15	1	25
4413	45	16	0	-25
4414	45	17	0	25
4415	45	18	0	25
4416	45	19	1	-25
4417	45	20	0	-25
4418	45	21	1	25
4419	45	22	1	-25
4420	45	23	1	25
4421	45	24	1	-25
4422	45	25	0	25
4423	45	26	0	25

Observation	Subject	Trial	Correct	Pressure
4424	45	27	0	-25
4425	45	28	1	25
4426	45	29	0	-25
4427	45	30	0	25
4428	45	31	1	-25
4429	45	32	1	25
4430	45	33	1	-25
4431	45	34	1	-25
4432	45	35	1	25
4433	45	36	1	25
4434	45	37	1	-25
4435	45	38	1	25
4436	45	39	1	-25
4437	45	40	1	-25
4438	45	41	0	25
4439	45	42	0	-25
4440	45	43	1	25
4441	45	44	0	-25
4442	45	45	0	25
4443	45	46	0	25
4444	45	47	0	-25
4445	45	48	1	25
4446	45	49	1	-25
4447	45	50	0	25
4448	45	51	0	0
4449	45	52	1	0
4450	45	53	1	0
4451	45	54	1	0
4452	45	55	0	0
4453	45	56	1	0
4454	45	57	1	0
4455	45	58	1	0
4456	45	59	0	0
4457	45	60	0	0
4458	45	61	1	0
4459	45	62	1	0
4460	45	63	1	0
4461	45	64	0	0
4462	45	65	1	0
4463	45	66	0	0
4464	45	67	1	0
4465	45	68	1	0
4466	45	69	1	0
4467	45	70	0	0

Observation	Subject	Trial	Correct	Pressure
4468	45	71	1	0
4469	45	72	1	0
4470	45	73	1	0
4471	45	74	0	0
4472	45	75	0	0
4473	45	76	0	0
4474	45	77	1	0
4475	45	78	1	0
4476	45	79	0	0
4477	45	80	1	0
4478	45	81	0	0
4479	45	82	1	0
4480	45	83	1	0
4481	45	84	1	0
4482	45	85	0	0
4483	45	86	1	0
4484	45	87	1	0
4485	45	88	1	0
4486	45	89	1	0
4487	45	90	0	0
4488	45	91	0	0
4489	45	92	1	0
4490	45	93	1	0
4491	45	94	0	0
4492	45	95	1	0
4493	45	96	1	0
4494	45	97	0	0
4495	45	98	0	0
4496	45	99	1	0
4497	45	100	0	0
4498	46	1	1	-25
4499	46	2	1	25
4500	46	3	1	-25
4501	46	4	1	-25
4502	46	5	0	25
4503	46	6	1	-25
4504	46	7	1	25
4505	46	8	1	-25
4506	46	9	1	25
4507	46	10	1	25
4508	46	11	1	-25
4509	46	12	0	25
4510	46	13	1	-25
4511	46	14	0	-25

Observation	Subject	Trial	Correct	Pressure
4512	46	15	1	25
4513	46	16	1	-25
4514	46	17	0	25
4515	46	18	1	25
4516	46	19	1	-25
4517	46	20	0	-25
4518	46	21	0	25
4519	46	22	1	-25
4520	46	23	1	25
4521	46	24	1	-25
4522	46	25	0	25
4523	46	26	0	25
4524	46	27	0	-25
4525	46	28	1	25
4526	46	29	0	-25
4527	46	30	1	25
4528	46	31	1	-25
4529	46	32	1	25
4530	46	33	1	-25
4531	46	34	1	-25
4532	46	35	1	25
4533	46	36	0	25
4534	46	37	1	-25
4535	46	38	0	25
4536	46	39	1	-25
4537	46	40	1	-25
4538	46	41	1	25
4539	46	42	1	-25
4540	46	43	1	25
4541	46	44	1	-25
4542	46	45	1	25
4543	46	46	0	25
4544	46	47	1	-25
4545	46	48	1	25
4546	46	49	0	-25
4547	46	50	0	25
4548	46	51	1	0
4549	46	52	0	0
4550	46	53	1	0
4551	46	54	1	0
4552	46	55	1	0
4553	46	56	1	0
4554	46	57	1	0
4555	46	58	0	0

Observation	Subject	Trial	Correct	Pressure
4556	46	59	0	0
4557	46	60	1	0
4558	46	61	1	0
4559	46	62	1	0
4560	46	63	1	0
4561	46	64	1	0
4562	46	65	1	0
4563	46	66	0	0
4564	46	67	0	0
4565	46	68	1	0
4566	46	69	1	0
4567	46	70	1	0
4568	46	71	1	0
4569	46	72	0	0
4570	46	73	0	0
4571	46	74	0	0
4572	46	75	1	0
4573	46	76	0	0
4574	46	77	1	0
4575	46	78	1	0
4576	46	79	0	0
4577	46	80	1	0
4578	46	81	0	0
4579	46	82	1	0
4580	46	83	1	0
4581	46	84	1	0
4582	46	85	1	0
4583	46	86	1	0
4584	46	87	0	0
4585	46	88	0	0
4586	46	89	1	0
4587	46	90	0	0
4588	46	91	1	0
4589	46	92	1	0
4590	46	93	1	0
4591	46	94	0	0
4592	46	95	1	0
4593	46	96	1	0
4594	46	97	0	0
4595	46	98	1	0
4596	46	99	1	0
4597	46	100	0	0
4598	47	1	1	-25
4599	47	2	0	25

Observation	Subject	Trial	Correct	Pressure
4600	47	3	1	-25
4601	47	4	1	-25
4602	47	5	0	25
4603	47	6	1	-25
4604	47	7	1	25
4605	47	8	1	-25
4606	47	9	1	25
4607	47	10	0	25
4608	47	11	1	-25
4609	47	12	0	25
4610	47	13	1	-25
4611	47	14	0	-25
4612	47	15	1	25
4613	47	16	1	-25
4614	47	17	0	25
4615	47	18	1	25
4616	47	19	1	-25
4617	47	20	1	-25
4618	47	21	1	25
4619	47	22	0	-25
4620	47	23	1	25
4621	47	24	0	-25
4622	47	25	0	25
4623	47	26	0	25
4624	47	27	0	-25
4625	47	28	1	25
4626	47	29	0	-25
4627	47	30	0	25
4628	47	31	1	-25
4629	47	32	0	25
4630	47	33	0	-25
4631	47	34	1	-25
4632	47	35	1	25
4633	47	36	1	25
4634	47	37	1	-25
4635	47	38	0	25
4636	47	39	0	-25
4637	47	40	0	-25
4638	47	41	1	25
4639	47	42	0	-25
4640	47	43	1	25
4641	47	44	0	-25
4642	47	45	1	25
4643	47	46	0	25

Observation	Subject	Trial	Correct	Pressure
4644	47	47	1	-25
4645	47	48	1	25
4646	47	49	0	-25
4647	47	50	1	25
4648	47	51	0	0
4649	47	52	1	0
4650	47	53	1	0
4651	47	54	0	0
4652	47	55	0	0
4653	47	56	1	0
4654	47	57	1	0
4655	47	58	0	0
4656	47	59	0	0
4657	47	60	0	0
4658	47	61	1	0
4659	47	62	1	0
4660	47	63	1	0
4661	47	64	0	0
4662	47	65	1	0
4663	47	66	0	0
4664	47	67	1	0
4665	47	68	1	0
4666	47	69	1	0
4667	47	70	0	0
4668	47	71	1	0
4669	47	72	0	0
4670	47	73	0	0
4671	47	74	0	0
4672	47	75	0	0
4673	47	76	0	0
4674	47	77	1	0
4675	47	78	1	0
4676	47	79	0	0
4677	47	80	1	0
4678	47	81	0	0
4679	47	82	1	0
4680	47	83	1	0
4681	47	84	1	0
4682	47	85	1	0
4683	47	86	1	0
4684	47	87	0	0
4685	47	88	0	0
4686	47	89	1	0
4687	47	90	0	0

Observation	Subject	Trial	Correct	Pressure
4688	47	91	0	0
4689	47	92	1	0
4690	47	93	1	0
4691	47	94	0	0
4692	47	95	1	0
4693	47	96	1	0
4694	47	97	1	0
4695	47	98	1	0
4696	47	99	1	0
4697	47	100	1	0
4698	48	1	1	-25
4699	48	2	1	25
4700	48	3	0	-25
4701	48	4	1	-25
4702	48	5	1	25
4703	48	6	0	-25
4704	48	7	1	25
4705	48	8	1	-25
4706	48	9	1	25
4707	48	10	1	25
4708	48	11	1	-25
4709	48	12	0	25
4710	48	13	1	-25
4711	48	14	0	-25
4712	48	15	1	25
4713	48	16	1	-25
4714	48	17	0	25
4715	48	18	1	25
4716	48	19	1	-25
4717	48	20	0	-25
4718	48	21	0	25
4719	48	22	1	-25
4720	48	23	1	25
4721	48	24	1	-25
4722	48	25	0	25
4723	48	26	0	25
4724	48	27	1	-25
4725	48	28	1	25
4726	48	29	1	-25
4727	48	30	0	25
4728	48	31	1	-25
4729	48	32	0	25
4730	48	33	0	-25
4731	48	34	1	-25

Observation	Subject	Trial	Correct	Pressure
4732	48	35	1	25
4733	48	36	0	25
4734	48	37	1	-25
4735	48	38	0	25
4736	48	39	1	-25
4737	48	40	1	-25
4738	48	41	1	25
4739	48	42	0	-25
4740	48	43	1	25
4741	48	44	1	-25
4742	48	45	0	25
4743	48	46	1	25
4744	48	47	1	-25
4745	48	48	1	25
4746	48	49	1	-25
4747	48	50	0	25
4748	48	51	1	0
4749	48	52	1	0
4750	48	53	1	0
4751	48	54	1	0
4752	48	55	0	0
4753	48	56	0	0
4754	48	57	1	0
4755	48	58	0	0
4756	48	59	1	0
4757	48	60	0	0
4758	48	61	1	0
4759	48	62	1	0
4760	48	63	1	0
4761	48	64	1	0
4762	48	65	0	0
4763	48	66	0	0
4764	48	67	1	0
4765	48	68	0	0
4766	48	69	1	0
4767	48	70	0	0
4768	48	71	1	0
4769	48	72	0	0
4770	48	73	1	0
4771	48	74	1	0
4772	48	75	1	0
4773	48	76	0	0
4774	48	77	1	0
4775	48	78	1	0

Observation	Subject	Trial	Correct	Pressure
4776	48	79	0	0
4777	48	80	1	0
4778	48	81	0	0
4779	48	82	1	0
4780	48	83	1	0
4781	48	84	1	0
4782	48	85	0	0
4783	48	86	1	0
4784	48	87	0	0
4785	48	88	0	0
4786	48	89	0	0
4787	48	90	0	0
4788	48	91	0	0
4789	48	92	0	0
4790	48	93	1	0
4791	48	94	0	0
4792	48	95	1	0
4793	48	96	1	0
4794	48	97	0	0
4795	48	98	0	0
4796	48	99	1	0
4797	48	100	0	0
4798	49	1	0	0
4799	49	2	1	0
4800	49	3	1	0
4801	49	4	1	0
4802	49	5	0	0
4803	49	6	0	0
4804	49	7	1	0
4805	49	8	0	0
4806	49	9	1	0
4807	49	10	1	0
4808	49	11	1	0
4809	49	12	0	0
4810	49	13	1	0
4811	49	14	1	0
4812	49	15	1	0
4813	49	16	1	0
4814	49	17	0	0
4815	49	18	1	0
4816	49	19	1	0
4817	49	20	0	0
4818	49	21	1	0
4819	49	22	1	0

Observation	Subject	Trial	Correct	Pressure
4820	49	23	0	0
4821	49	24	1	0
4822	49	25	0	0
4823	49	26	0	0
4824	49	27	0	0
4825	49	28	1	0
4826	49	29	0	0
4827	49	30	0	0
4828	49	31	1	0
4829	49	32	1	0
4830	49	33	1	0
4831	49	34	1	0
4832	49	35	1	0
4833	49	36	1	0
4834	49	37	1	0
4835	49	38	0	0
4836	49	39	1	0
4837	49	40	0	0
4838	49	41	1	0
4839	49	42	0	0
4840	49	43	1	0
4841	49	44	1	0
4842	49	45	1	0
4843	49	46	0	0
4844	49	47	1	0
4845	49	48	1	0
4846	49	49	0	0
4847	49	50	0	0
4848	49	51	1	-25
4849	49	52	0	25
4850	49	53	0	-25
4851	49	54	1	-25
4852	49	55	0	25
4853	49	56	0	-25
4854	49	57	1	25
4855	49	58	1	-25
4856	49	59	0	25
4857	49	60	1	25
4858	49	61	1	-25
4859	49	62	1	25
4860	49	63	1	-25
4861	49	64	1	-25
4862	49	65	0	25
4863	49	66	0	-25

Observation	Subject	Trial	Correct	Pressure
4864	49	67	1	25
4865	49	68	0	25
4866	49	69	1	-25
4867	49	70	0	-25
4868	49	71	1	25
4869	49	72	0	-25
4870	49	73	1	25
4871	49	74	0	-25
4872	49	75	1	25
4873	49	76	0	25
4874	49	77	1	-25
4875	49	78	1	25
4876	49	79	0	-25
4877	49	80	1	25
4878	49	81	0	-25
4879	49	82	1	25
4880	49	83	1	-25
4881	49	84	1	-25
4882	49	85	1	25
4883	49	86	1	25
4884	49	87	0	-25
4885	49	88	1	25
4886	49	89	0	-25
4887	49	90	1	-25
4888	49	91	1	25
4889	49	92	0	-25
4890	49	93	1	25
4891	49	94	0	-25
4892	49	95	1	25
4893	49	96	0	25
4894	49	97	0	-25
4895	49	98	1	25
4896	49	99	1	-25
4897	49	100	0	25
4898	50	1	1	0
4899	50	2	0	0
4900	50	3	1	0
4901	50	4	1	0
4902	50	5	0	0
4903	50	6	1	0
4904	50	7	1	0
4905	50	8	1	0
4906	50	9	1	0
4907	50	10	1	0

Observation	Subject	Trial	Correct	Pressure
4908	50	11	1	0
4909	50	12	0	0
4910	50	13	0	0
4911	50	14	0	0
4912	50	15	1	0
4913	50	16	0	0
4914	50	17	1	0
4915	50	18	1	0
4916	50	19	1	0
4917	50	20	1	0
4918	50	21	1	0
4919	50	22	1	0
4920	50	23	0	0
4921	50	24	1	0
4922	50	25	1	0
4923	50	26	1	0
4924	50	27	0	0
4925	50	28	1	0
4926	50	29	0	0
4927	50	30	0	0
4928	50	31	1	0
4929	50	32	1	0
4930	50	33	0	0
4931	50	34	1	0
4932	50	35	1	0
4933	50	36	1	0
4934	50	37	1	0
4935	50	38	0	0
4936	50	39	1	0
4937	50	40	1	0
4938	50	41	0	0
4939	50	42	1	0
4940	50	43	0	0
4941	50	44	0	0
4942	50	45	1	0
4943	50	46	0	0
4944	50	47	0	0
4945	50	48	1	0
4946	50	49	1	0
4947	50	50	0	0
4948	50	51	1	-25
4949	50	52	0	25
4950	50	53	0	-25
4951	50	54	0	-25

Observation	Subject	Trial	Correct	Pressure
4952	50	55	0	25
4953	50	56	1	-25
4954	50	57	1	25
4955	50	58	0	-25
4956	50	59	0	25
4957	50	60	1	25
4958	50	61	1	-25
4959	50	62	1	25
4960	50	63	1	-25
4961	50	64	0	-25
4962	50	65	1	25
4963	50	66	0	-25
4964	50	67	1	25
4965	50	68	1	25
4966	50	69	1	-25
4967	50	70	1	-25
4968	50	71	1	25
4969	50	72	0	-25
4970	50	73	0	25
4971	50	74	0	-25
4972	50	75	0	25
4973	50	76	1	25
4974	50	77	1	-25
4975	50	78	0	25
4976	50	79	0	-25
4977	50	80	1	25
4978	50	81	0	-25
4979	50	82	1	25
4980	50	83	1	-25
4981	50	84	1	-25
4982	50	85	0	25
4983	50	86	1	25
4984	50	87	1	-25
4985	50	88	0	25
4986	50	89	0	-25
4987	50	90	1	-25
4988	50	91	1	25
4989	50	92	0	-25
4990	50	93	1	25
4991	50	94	0	-25
4992	50	95	1	25
4993	50	96	1	25
4994	50	97	1	-25
4995	50	98	0	25

Observation	Subject	Trial	Correct	Pressure
4996	50	99	1	-25
4997	50	100	0	25
4998	51	1	1	0
4999	51	2	1	0
5000	51	3	1	0
5001	51	4	1	0
5002	51	5	1	0
5003	51	6	1	0
5004	51	7	1	0
5005	51	8	1	0
5006	51	9	1	0
5007	51	10	0	0
5008	51	11	1	0
5009	51	12	1	0
5010	51	13	1	0
5011	51	14	1	0
5012	51	15	1	0
5013	51	16	1	0
5014	51	17	0	0
5015	51	18	1	0
5016	51	19	1	0
5017	51	20	0	0
5018	51	21	1	0
5019	51	22	1	0
5020	51	23	1	0
5021	51	24	1	0
5022	51	25	0	0
5023	51	26	0	0
5024	51	27	0	0
5025	51	28	1	0
5026	51	29	1	0
5027	51	30	0	0
5028	51	31	1	0
5029	51	32	1	0
5030	51	33	1	0
5031	51	34	1	0
5032	51	35	1	0
5033	51	36	1	0
5034	51	37	1	0
5035	51	38	0	0
5036	51	39	1	0
5037	51	40	1	0
5038	51	41	1	0
5039	51	42	1	0

Observation	Subject	Trial	Correct	Pressure
5040	51	43	1	0
5041	51	44	0	0
5042	51	45	1	0
5043	51	46	1	0
5044	51	47	1	0
5045	51	48	1	0
5046	51	49	1	0
5047	51	50	1	0
5048	51	51	1	-25
5049	51	52	1	25
5050	51	53	1	-25
5051	51	54	1	-25
5052	51	55	1	25
5053	51	56	1	-25
5054	51	57	1	25
5055	51	58	0	-25
5056	51	59	0	25
5057	51	60	1	25
5058	51	61	1	-25
5059	51	62	1	25
5060	51	63	1	-25
5061	51	64	1	-25
5062	51	65	1	25
5063	51	66	1	-25
5064	51	67	1	25
5065	51	68	0	25
5066	51	69	1	-25
5067	51	70	1	-25
5068	51	71	1	25
5069	51	72	1	-25
5070	51	73	0	25
5071	51	74	0	-25
5072	51	75	0	25
5073	51	76	1	25
5074	51	77	1	-25
5075	51	78	1	25
5076	51	79	0	-25
5077	51	80	1	25
5078	51	81	0	-25
5079	51	82	1	25
5080	51	83	1	-25
5081	51	84	0	-25
5082	51	85	0	25
5083	51	86	1	25

Observation	Subject	Trial	Correct	Pressure
5084	51	87	0	-25
5085	51	88	1	25
5086	51	89	0	-25
5087	51	90	1	-25
5088	51	91	1	25
5089	51	92	0	-25
5090	51	93	1	25
5091	51	94	0	-25
5092	51	95	1	25
5093	51	96	1	25
5094	51	97	0	-25
5095	51	98	0	25
5096	51	99	1	-25
5097	51	100	0	25
5098	52	1	1	0
5099	52	2	0	0
5100	52	3	1	0
5101	52	4	1	0
5102	52	5	0	0
5103	52	6	1	0
5104	52	7	1	0
5105	52	8	0	0
5106	52	9	1	0
5107	52	10	1	0
5108	52	11	1	0
5109	52	12	0	0
5110	52	13	0	0
5111	52	14	1	0
5112	52	15	1	0
5113	52	16	1	0
5114	52	17	0	0
5115	52	18	1	0
5116	52	19	1	0
5117	52	20	0	0
5118	52	21	1	0
5119	52	22	1	0
5120	52	23	1	0
5121	52	24	1	0
5122	52	25	1	0
5123	52	26	0	0
5124	52	27	0	0
5125	52	28	1	0
5126	52	29	0	0
5127	52	30	1	0

Observation	Subject	Trial	Correct	Pressure
5128	52	31	1	0
5129	52	32	1	0
5130	52	33	0	0
5131	52	34	1	0
5132	52	35	1	0
5133	52	36	1	0
5134	52	37	1	0
5135	52	38	0	0
5136	52	39	0	0
5137	52	40	1	0
5138	52	41	1	0
5139	52	42	1	0
5140	52	43	1	0
5141	52	44	1	0
5142	52	45	0	0
5143	52	46	1	0
5144	52	47	1	0
5145	52	48	1	0
5146	52	49	1	0
5147	52	50	1	0
5148	52	51	1	-25
5149	52	52	1	25
5150	52	53	1	-25
5151	52	54	1	-25
5152	52	55	0	25
5153	52	56	0	-25
5154	52	57	1	25
5155	52	58	1	-25
5156	52	59	0	25
5157	52	60	0	25
5158	52	61	1	-25
5159	52	62	1	25
5160	52	63	1	-25
5161	52	64	1	-25
5162	52	65	1	25
5163	52	66	0	-25
5164	52	67	0	25
5165	52	68	1	25
5166	52	69	1	-25
5167	52	70	0	-25
5168	52	71	1	25
5169	52	72	0	-25
5170	52	73	1	25
5171	52	74	0	-25

Observation	Subject	Trial	Correct	Pressure
5172	52	75	1	25
5173	52	76	0	25
5174	52	77	0	-25
5175	52	78	1	25
5176	52	79	0	-25
5177	52	80	1	25
5178	52	81	0	-25
5179	52	82	1	25
5180	52	83	1	-25
5181	52	84	1	-25
5182	52	85	0	25
5183	52	86	1	25
5184	52	87	1	-25
5185	52	88	1	25
5186	52	89	0	-25
5187	52	90	0	-25
5188	52	91	1	25
5189	52	92	0	-25
5190	52	93	1	25
5191	52	94	0	-25
5192	52	95	1	25
5193	52	96	1	25
5194	52	97	1	-25
5195	52	98	0	25
5196	52	99	1	-25
5197	52	100	1	25
5198	53	1	1	-25
5199	53	2	0	25
5200	53	3	1	-25
5201	53	4	1	-25
5202	53	5	0	25
5203	53	6	1	-25
5204	53	7	1	25
5205	53	8	1	-25
5206	53	9	1	25
5207	53	10	1	25
5208	53	11	1	-25
5209	53	12	0	25
5210	53	13	1	-25
5211	53	14	0	-25
5212	53	15	1	25
5213	53	16	1	-25
5214	53	17	1	25
5215	53	18	1	25

Observation	Subject	Trial	Correct	Pressure
5216	53	19	1	-25
5217	53	20	0	-25
5218	53	21	1	25
5219	53	22	1	-25
5220	53	23	0	25
5221	53	24	1	-25
5222	53	25	1	25
5223	53	26	0	25
5224	53	27	0	-25
5225	53	28	1	25
5226	53	29	1	-25
5227	53	30	0	25
5228	53	31	1	-25
5229	53	32	0	25
5230	53	33	0	-25
5231	53	34	1	-25
5232	53	35	0	25
5233	53	36	0	25
5234	53	37	1	-25
5235	53	38	0	25
5236	53	39	0	-25
5237	53	40	1	-25
5238	53	41	0	25
5239	53	42	1	-25
5240	53	43	1	25
5241	53	44	1	-25
5242	53	45	1	25
5243	53	46	0	25
5244	53	47	0	-25
5245	53	48	1	25
5246	53	49	0	-25
5247	53	50	0	25
5248	53	51	1	0
5249	53	52	1	0
5250	53	53	1	0
5251	53	54	1	0
5252	53	55	1	0
5253	53	56	1	0
5254	53	57	1	0
5255	53	58	0	0
5256	53	59	0	0
5257	53	60	0	0
5258	53	61	1	0
5259	53	62	1	0

Observation	Subject	Trial	Correct	Pressure
5260	53	63	1	0
5261	53	64	0	0
5262	53	65	0	0
5263	53	66	1	0
5264	53	67	1	0
5265	53	68	0	0
5266	53	69	1	0
5267	53	70	0	0
5268	53	71	1	0
5269	53	72	0	0
5270	53	73	1	0
5271	53	74	0	0
5272	53	75	1	0
5273	53	76	1	0
5274	53	77	1	0
5275	53	78	1	0
5276	53	79	0	0
5277	53	80	1	0
5278	53	81	0	0
5279	53	82	1	0
5280	53	83	1	0
5281	53	84	1	0
5282	53	85	1	0
5283	53	86	1	0
5284	53	87	1	0
5285	53	88	0	0
5286	53	89	0	0
5287	53	90	0	0
5288	53	91	0	0
5289	53	92	1	0
5290	53	93	1	0
5291	53	94	1	0
5292	53	95	1	0
5293	53	96	1	0
5294	53	97	1	0
5295	53	98	1	0
5296	53	99	1	0
5297	53	100	0	0
5298	54	1	1	-25
5299	54	2	1	25
5300	54	3	1	-25
5301	54	4	1	-25
5302	54	5	0	25
5303	54	6	0	-25

Observation	Subject	Trial	Correct	Pressure
5304	54	7	1	25
5305	54	8	0	-25
5306	54	9	1	25
5307	54	10	1	25
5308	54	11	1	-25
5309	54	12	1	25
5310	54	13	1	-25
5311	54	14	0	-25
5312	54	15	1	25
5313	54	16	1	-25
5314	54	17	1	25
5315	54	18	1	25
5316	54	19	1	-25
5317	54	20	0	-25
5318	54	21	1	25
5319	54	22	1	-25
5320	54	23	1	25
5321	54	24	1	-25
5322	54	25	0	25
5323	54	26	1	25
5324	54	27	1	-25
5325	54	28	1	25
5326	54	29	1	-25
5327	54	30	0	25
5328	54	31	1	-25
5329	54	32	1	25
5330	54	33	0	-25
5331	54	34	1	-25
5332	54	35	0	25
5333	54	36	0	25
5334	54	37	1	-25
5335	54	38	0	25
5336	54	39	0	-25
5337	54	40	1	-25
5338	54	41	1	25
5339	54	42	1	-25
5340	54	43	1	25
5341	54	44	0	-25
5342	54	45	1	25
5343	54	46	1	25
5344	54	47	0	-25
5345	54	48	1	25
5346	54	49	1	-25
5347	54	50	0	25

Observation	Subject	Trial	Correct	Pressure
5348	54	51	0	0
5349	54	52	0	0
5350	54	53	1	0
5351	54	54	1	0
5352	54	55	0	0
5353	54	56	0	0
5354	54	57	1	0
5355	54	58	0	0
5356	54	59	1	0
5357	54	60	0	0
5358	54	61	1	0
5359	54	62	1	0
5360	54	63	1	0
5361	54	64	0	0
5362	54	65	0	0
5363	54	66	0	0
5364	54	67	0	0
5365	54	68	0	0
5366	54	69	1	0
5367	54	70	0	0
5368	54	71	1	0
5369	54	72	1	0
5370	54	73	0	0
5371	54	75	1	0
5372	54	76	1	0
5373	54	77	1	0
5374	54	78	1	0
5375	54	79	0	0
5376	54	80	1	0
5377	54	81	0	0
5378	54	82	1	0
5379	54	83	1	0
5380	54	84	1	0
5381	54	85	0	0
5382	54	86	1	0
5383	54	87	0	0
5384	54	88	1	0
5385	54	89	0	0
5386	54	90	0	0
5387	54	91	1	0
5388	54	92	1	0
5389	54	93	1	0
5390	54	94	0	0
5391	54	95	0	0

Observation	Subject	Trial	Correct	Pressure
5392	54	96	1	0
5393	54	97	0	0
5394	54	98	0	0
5395	54	99	0	0
5396	54	100	0	0
5397	55	1	1	-25
5398	55	2	1	25
5399	55	3	1	-25
5400	55	4	1	-25
5401	55	5	0	25
5402	55	6	1	-25
5403	55	7	1	25
5404	55	8	0	-25
5405	55	9	1	25
5406	55	10	1	25
5407	55	11	1	-25
5408	55	12	0	25
5409	55	13	1	-25
5410	55	14	0	-25
5411	55	15	1	25
5412	55	16	1	-25
5413	55	17	0	25
5414	55	18	1	25
5415	55	19	0	-25
5416	55	20	0	-25
5417	55	21	1	25
5418	55	22	0	-25
5419	55	23	1	25
5420	55	24	1	-25
5421	55	25	0	25
5422	55	26	1	25
5423	55	27	0	-25
5424	55	28	1	25
5425	55	29	1	-25
5426	55	30	0	25
5427	55	31	1	-25
5428	55	32	0	25
5429	55	33	1	-25
5430	55	34	1	-25
5431	55	35	0	25
5432	55	36	1	25
5433	55	37	1	-25
5434	55	38	0	25
5435	55	39	0	-25

Observation	Subject	Trial	Correct	Pressure
5436	55	40	1	-25
5437	55	41	1	25
5438	55	42	0	-25
5439	55	43	1	25
5440	55	44	0	-25
5441	55	45	1	25
5442	55	46	0	25
5443	55	47	1	-25
5444	55	48	1	25
5445	55	49	1	-25
5446	55	50	0	25
5447	55	51	1	0
5448	55	52	0	0
5449	55	53	1	0
5450	55	54	0	0
5451	55	55	0	0
5452	55	56	0	0
5453	55	57	1	0
5454	55	58	0	0
5455	55	59	1	0
5456	55	60	1	0
5457	55	61	1	0
5458	55	62	1	0
5459	55	63	1	0
5460	55	64	0	0
5461	55	65	1	0
5462	55	66	0	0
5463	55	67	1	0
5464	55	68	0	0
5465	55	69	1	0
5466	55	70	1	0
5467	55	71	1	0
5468	55	72	0	0
5469	55	73	1	0
5470	55	74	0	0
5471	55	75	0	0
5472	55	76	0	0
5473	55	77	1	0
5474	55	78	1	0
5475	55	79	1	0
5476	55	80	1	0
5477	55	81	1	0
5478	55	82	1	0
5479	55	83	1	0

Observation	Subject	Trial	Correct	Pressure
5480	55	84	1	0
5481	55	85	1	0
5482	55	86	1	0
5483	55	87	1	0
5484	55	88	1	0
5485	55	89	0	0
5486	55	90	0	0
5487	55	91	1	0
5488	55	92	1	0
5489	55	93	1	0
5490	55	94	0	0
5491	55	95	1	0
5492	55	96	1	0
5493	55	97	0	0
5494	55	98	0	0
5495	55	99	1	0
5496	55	100	0	0
5497	56	1	1	-25
5498	56	2	1	25
5499	56	3	1	-25
5500	56	4	1	-25
5501	56	5	0	25
5502	56	6	1	-25
5503	56	7	1	25
5504	56	8	0	-25
5505	56	9	1	25
5506	56	10	1	25
5507	56	11	1	-25
5508	56	12	1	25
5509	56	13	1	-25
5510	56	14	0	-25
5511	56	15	1	25
5512	56	16	0	-25
5513	56	17	0	25
5514	56	18	1	25
5515	56	19	1	-25
5516	56	20	0	-25
5517	56	21	1	25
5518	56	22	0	-25
5519	56	23	0	25
5520	56	24	1	-25
5521	56	25	0	25
5522	56	26	0	25
5523	56	27	0	-25

Observation	Subject	Trial	Correct	Pressure
5524	56	28	1	25
5525	56	29	0	-25
5526	56	30	0	25
5527	56	31	1	-25
5528	56	32	0	25
5529	56	33	0	-25
5530	56	34	1	-25
5531	56	35	0	25
5532	56	36	0	25
5533	56	37	1	-25
5534	56	38	0	25
5535	56	39	0	-25
5536	56	40	1	-25
5537	56	41	1	25
5538	56	42	0	-25
5539	56	43	1	25
5540	56	44	1	-25
5541	56	45	1	25
5542	56	46	0	25
5543	56	47	0	-25
5544	56	48	1	25
5545	56	49	0	-25
5546	56	50	0	25
5547	56	51	0	0
5548	56	52	1	0
5549	56	53	1	0
5550	56	54	1	0
5551	56	55	0	0
5552	56	56	0	0
5553	56	57	1	0
5554	56	58	0	0
5555	56	59	0	0
5556	56	60	0	0
5557	56	61	1	0
5558	56	62	1	0
5559	56	63	0	0
5560	56	64	0	0
5561	56	65	1	0
5562	56	66	1	0
5563	56	67	1	0
5564	56	68	1	0
5565	56	69	1	0
5566	56	70	0	0
5567	56	71	1	0

Observation	Subject	Trial	Correct	Pressure
5568	56	72	1	0
5569	56	73	1	0
5570	56	74	0	0
5571	56	75	0	0
5572	56	76	0	0
5573	56	77	1	0
5574	56	78	1	0
5575	56	79	0	0
5576	56	80	1	0
5577	56	81	1	0
5578	56	82	1	0
5579	56	83	1	0
5580	56	84	0	0
5581	56	85	0	0
5582	56	86	1	0
5583	56	87	1	0
5584	56	88	1	0
5585	56	89	0	0
5586	56	90	1	0
5587	56	91	1	0
5588	56	92	0	0
5589	56	93	1	0
5590	56	94	0	0
5591	56	95	1	0
5592	56	96	1	0
5593	56	97	0	0
5594	56	98	1	0
5595	56	99	1	0
5596	56	100	0	0
5597	57	1	1	0
5598	57	2	0	0
5599	57	3	1	0
5600	57	4	0	0
5601	57	5	0	0
5602	57	6	0	0
5603	57	7	1	0
5604	57	8	0	0
5605	57	9	1	0
5606	57	10	1	0
5607	57	11	1	0
5608	57	12	0	0
5609	57	13	1	0
5610	57	14	0	0
5611	57	15	1	0

Observation	Subject	Trial	Correct	Pressure
5612	57	16	0	0
5613	57	17	0	0
5614	57	18	1	0
5615	57	19	1	0
5616	57	20	0	0
5617	57	21	1	0
5618	57	22	0	0
5619	57	23	1	0
5620	57	24	1	0
5621	57	25	0	0
5622	57	26	0	0
5623	57	27	0	0
5624	57	28	1	0
5625	57	29	0	0
5626	57	30	0	0
5627	57	31	1	0
5628	57	32	0	0
5629	57	33	1	0
5630	57	34	1	0
5631	57	35	0	0
5632	57	36	1	0
5633	57	37	1	0
5634	57	38	0	0
5635	57	39	0	0
5636	57	40	1	0
5637	57	41	1	0
5638	57	42	1	0
5639	57	43	1	0
5640	57	44	0	0
5641	57	45	1	0
5642	57	46	1	0
5643	57	47	0	0
5644	57	48	1	0
5645	57	49	1	0
5646	57	50	0	0
5647	57	51	0	-25
5648	57	52	1	25
5649	57	53	1	-25
5650	57	54	1	-25
5651	57	55	0	25
5652	57	56	0	-25
5653	57	57	1	25
5654	57	58	0	-25
5655	57	59	0	25

Observation	Subject	Trial	Correct	Pressure
5656	57	60	1	25
5657	57	61	1	-25
5658	57	62	0	25
5659	57	63	1	-25
5660	57	64	0	-25
5661	57	65	0	25
5662	57	66	0	-25
5663	57	67	0	25
5664	57	68	0	25
5665	57	69	1	-25
5666	57	70	1	-25
5667	57	71	1	25
5668	57	72	0	-25
5669	57	73	1	25
5670	57	74	1	-25
5671	57	75	0	25
5672	57	76	1	25
5673	57	77	1	-25
5674	57	78	1	25
5675	57	79	0	-25
5676	57	80	1	25
5677	57	81	0	-25
5678	57	82	1	25
5679	57	83	1	-25
5680	57	84	1	-25
5681	57	85	1	25
5682	57	86	1	25
5683	57	87	0	-25
5684	57	88	1	25
5685	57	89	1	-25
5686	57	90	0	-25
5687	57	91	0	25
5688	57	92	0	-25
5689	57	93	1	25
5690	57	94	0	-25
5691	57	95	1	25
5692	57	96	1	25
5693	57	97	0	-25
5694	57	98	0	25
5695	57	99	0	-25
5696	57	100	0	25
5697	58	1	1	0
5698	58	2	0	0
5699	58	3	1	0

Observation	Subject	Trial	Correct	Pressure
5700	58	4	1	0
5701	58	5	0	0
5702	58	6	0	0
5703	58	7	1	0
5704	58	8	0	0
5705	58	9	1	0
5706	58	10	1	0
5707	58	11	1	0
5708	58	12	0	0
5709	58	13	0	0
5710	58	14	0	0
5711	58	15	1	0
5712	58	16	1	0
5713	58	17	1	0
5714	58	18	1	0
5715	58	19	1	0
5716	58	20	0	0
5717	58	21	0	0
5718	58	22	1	0
5719	58	23	0	0
5720	58	24	1	0
5721	58	25	1	0
5722	58	26	0	0
5723	58	27	0	0
5724	58	28	1	0
5725	58	29	0	0
5726	58	30	1	0
5727	58	31	1	0
5728	58	32	1	0
5729	58	33	0	0
5730	58	34	1	0
5731	58	35	1	0
5732	58	36	1	0
5733	58	37	1	0
5734	58	38	0	0
5735	58	39	1	0
5736	58	40	1	0
5737	58	41	1	0
5738	58	42	0	0
5739	58	43	1	0
5740	58	44	1	0
5741	58	45	1	0
5742	58	46	1	0
5743	58	47	1	0

Observation	Subject	Trial	Correct	Pressure
5744	58	48	1	0
5745	58	49	0	0
5746	58	50	1	0
5747	58	51	0	-25
5748	58	52	1	25
5749	58	53	1	-25
5750	58	54	1	-25
5751	58	55	1	25
5752	58	56	0	-25
5753	58	57	1	25
5754	58	58	0	-25
5755	58	59	1	25
5756	58	60	1	25
5757	58	61	1	-25
5758	58	62	1	25
5759	58	63	1	-25
5760	58	64	0	-25
5761	58	65	1	25
5762	58	66	0	-25
5763	58	67	1	25
5764	58	68	1	25
5765	58	69	1	-25
5766	58	70	1	-25
5767	58	71	1	25
5768	58	72	1	-25
5769	58	73	1	25
5770	58	74	0	-25
5771	58	75	0	25
5772	58	76	0	25
5773	58	77	0	-25
5774	58	78	1	25
5775	58	79	0	-25
5776	58	80	1	25
5777	58	81	0	-25
5778	58	82	1	25
5779	58	83	1	-25
5780	58	84	1	-25
5781	58	85	0	25
5782	58	86	1	25
5783	58	87	0	-25
5784	58	88	0	25
5785	58	89	0	-25
5786	58	90	1	-25
5787	58	91	0	25

Observation	Subject	Trial	Correct	Pressure
5788	58	92	0	-25
5789	58	93	1	25
5790	58	94	0	-25
5791	58	95	1	25
5792	58	96	1	25
5793	58	97	1	-25
5794	58	98	0	25
5795	58	99	1	-25
5796	58	100	1	25
5797	59	1	1	0
5798	59	2	1	0
5799	59	3	1	0
5800	59	4	1	0
5801	59	5	1	0
5802	59	6	1	0
5803	59	7	1	0
5804	59	8	0	0
5805	59	9	1	0
5806	59	10	0	0
5807	59	11	1	0
5808	59	12	0	0
5809	59	13	0	0
5810	59	14	0	0
5811	59	15	1	0
5812	59	16	0	0
5813	59	17	0	0
5814	59	18	1	0
5815	59	19	1	0
5816	59	20	0	0
5817	59	21	0	0
5818	59	22	0	0
5819	59	23	1	0
5820	59	24	1	0
5821	59	25	0	0
5822	59	26	1	0
5823	59	27	0	0
5824	59	28	1	0
5825	59	29	0	0
5826	59	30	0	0
5827	59	31	1	0
5828	59	32	0	0
5829	59	33	1	0
5830	59	34	1	0
5831	59	35	1	0

Observation	Subject	Trial	Correct	Pressure
5832	59	36	0	0
5833	59	37	1	0
5834	59	38	0	0
5835	59	39	1	0
5836	59	40	1	0
5837	59	41	1	0
5838	59	42	0	0
5839	59	43	0	0
5840	59	44	0	0
5841	59	45	1	0
5842	59	46	1	0
5843	59	47	1	0
5844	59	48	1	0
5845	59	49	1	0
5846	59	50	0	0
5847	59	51	0	-25
5848	59	52	1	25
5849	59	53	0	-25
5850	59	54	1	-25
5851	59	55	1	25
5852	59	56	0	-25
5853	59	57	1	25
5854	59	58	0	-25
5855	59	59	0	25
5856	59	60	0	25
5857	59	61	1	-25
5858	59	62	1	25
5859	59	63	1	-25
5860	59	64	1	-25
5861	59	65	1	25
5862	59	66	0	-25
5863	59	67	1	25
5864	59	68	1	25
5865	59	69	1	-25
5866	59	70	1	-25
5867	59	71	1	25
5868	59	72	1	-25
5869	59	73	1	25
5870	59	74	1	-25
5871	59	75	0	25
5872	59	76	1	25
5873	59	77	0	-25
5874	59	78	0	25
5875	59	79	0	-25

Observation	Subject	Trial	Correct	Pressure
5876	59	80	1	25
5877	59	81	1	-25
5878	59	82	1	25
5879	59	83	1	-25
5880	59	84	0	-25
5881	59	85	1	25
5882	59	86	1	25
5883	59	87	0	-25
5884	59	88	0	25
5885	59	89	0	-25
5886	59	90	1	-25
5887	59	91	0	25
5888	59	92	0	-25
5889	59	93	1	25
5890	59	94	0	-25
5891	59	95	1	25
5892	59	96	1	25
5893	59	97	1	-25
5894	59	98	1	25
5895	59	99	0	-25
5896	59	100	0	25
5897	60	1	0	0
5898	60	2	0	0
5899	60	3	1	0
5900	60	4	0	0
5901	60	5	0	0
5902	60	6	1	0
5903	60	7	1	0
5904	60	8	0	0
5905	60	9	1	0
5906	60	10	0	0
5907	60	11	1	0
5908	60	12	0	0
5909	60	13	1	0
5910	60	14	0	0
5911	60	15	1	0
5912	60	16	1	0
5913	60	17	1	0
5914	60	18	1	0
5915	60	19	1	0
5916	60	20	0	0
5917	60	21	1	0
5918	60	22	1	0
5919	60	23	1	0

Observation	Subject	Trial	Correct	Pressure
5920	60	24	1	0
5921	60	25	0	0
5922	60	26	1	0
5923	60	27	1	0
5924	60	28	1	0
5925	60	29	0	0
5926	60	30	0	0
5927	60	31	1	0
5928	60	32	1	0
5929	60	33	0	0
5930	60	34	1	0
5931	60	35	0	0
5932	60	36	0	0
5933	60	37	0	0
5934	60	38	0	0
5935	60	39	0	0
5936	60	40	1	0
5937	60	41	1	0
5938	60	42	1	0
5939	60	43	1	0
5940	60	44	1	0
5941	60	45	1	0
5942	60	46	0	0
5943	60	47	1	0
5944	60	48	1	0
5945	60	49	1	0
5946	60	50	0	0
5947	60	51	1	-25
5948	60	52	0	25
5949	60	53	1	-25
5950	60	54	1	-25
5951	60	55	0	25
5952	60	56	0	-25
5953	60	57	1	25
5954	60	58	0	-25
5955	60	59	1	25
5956	60	60	0	25
5957	60	61	1	-25
5958	60	62	1	25
5959	60	63	1	-25
5960	60	64	0	-25
5961	60	65	1	25
5962	60	66	0	-25
5963	60	67	1	25

Observation	Subject	Trial	Correct	Pressure
5964	60	68	1	25
5965	60	69	1	-25
5966	60	70	0	-25
5967	60	71	1	25
5968	60	72	0	-25
5969	60	73	1	25
5970	60	74	0	-25
5971	60	75	0	25
5972	60	76	1	25
5973	60	77	0	-25
5974	60	78	1	25
5975	60	79	1	-25
5976	60	80	1	25
5977	60	81	1	-25
5978	60	82	0	25
5979	60	83	1	-25
5980	60	84	0	-25
5981	60	85	0	25
5982	60	86	1	25
5983	60	87	0	-25
5984	60	88	0	25
5985	60	89	0	-25
5986	60	90	0	-25
5987	60	91	1	25
5988	60	92	0	-25
5989	60	93	1	25
5990	60	94	0	-25
5991	60	95	0	25
5992	60	96	1	25
5993	60	97	1	-25
5994	60	98	1	25
5995	60	99	0	-25
5996	60	100	1	25
5997	61	1	1	-25
5998	61	2	0	25
5999	61	3	0	-25
6000	61	4	1	-25
6001	61	5	0	25
6002	61	6	0	-25
6003	61	7	1	25
6004	61	8	1	-25
6005	61	9	1	25
6006	61	10	1	25
6007	61	11	1	-25

Observation	Subject	Trial	Correct	Pressure
6008	61	12	0	25
6009	61	13	0	-25
6010	61	14	0	-25
6011	61	15	1	25
6012	61	16	0	-25
6013	61	17	0	25
6014	61	18	1	25
6015	61	19	1	-25
6016	61	20	0	-25
6017	61	21	0	25
6018	61	22	1	-25
6019	61	23	1	25
6020	61	24	1	-25
6021	61	25	0	25
6022	61	26	0	25
6023	61	27	0	-25
6024	61	28	1	25
6025	61	29	0	-25
6026	61	30	0	25
6027	61	31	1	-25
6028	61	32	1	25
6029	61	33	0	-25
6030	61	34	1	-25
6031	61	35	0	25
6032	61	36	1	25
6033	61	37	1	-25
6034	61	38	0	25
6035	61	39	1	-25
6036	61	40	0	-25
6037	61	41	0	25
6038	61	42	0	-25
6039	61	43	1	25
6040	61	44	0	-25
6041	61	45	1	25
6042	61	46	0	25
6043	61	47	0	-25
6044	61	48	1	25
6045	61	49	1	-25
6046	61	50	0	25
6047	61	51	0	0
6048	61	52	1	0
6049	61	53	1	0
6050	61	54	1	0
6051	61	55	0	0

Observation	Subject	Trial	Correct	Pressure
6052	61	56	0	0
6053	61	57	1	0
6054	61	58	0	0
6055	61	59	1	0
6056	61	60	0	0
6057	61	61	1	0
6058	61	62	1	0
6059	61	63	1	0
6060	61	64	0	0
6061	61	65	0	0
6062	61	66	0	0
6063	61	67	1	0
6064	61	68	0	0
6065	61	69	1	0
6066	61	70	1	0
6067	61	71	1	0
6068	61	72	0	0
6069	61	73	1	0
6070	61	74	0	0
6071	61	75	0	0
6072	61	76	0	0
6073	61	77	1	0
6074	61	78	1	0
6075	61	79	0	0
6076	61	80	1	0
6077	61	81	1	0
6078	61	82	1	0
6079	61	83	1	0
6080	61	84	1	0
6081	61	85	0	0
6082	61	86	1	0
6083	61	87	1	0
6084	61	88	1	0
6085	61	89	1	0
6086	61	90	0	0
6087	61	91	1	0
6088	61	92	0	0
6089	61	93	1	0
6090	61	94	1	0
6091	61	95	1	0
6092	61	96	1	0
6093	61	97	0	0
6094	61	98	0	0
6095	61	99	1	0

Observation	Subject	Trial	Correct	Pressure
6096	61	100	0	0
6097	62	1	1	-25
6098	62	2	0	25
6099	62	3	1	-25
6100	62	4	1	-25
6101	62	5	1	25
6102	62	6	1	-25
6103	62	7	1	25
6104	62	8	1	-25
6105	62	9	1	25
6106	62	10	1	25
6107	62	11	1	-25
6108	62	12	1	25
6109	62	13	1	-25
6110	62	14	0	-25
6111	62	15	1	25
6112	62	16	0	-25
6113	62	17	0	25
6114	62	18	1	25
6115	62	19	1	-25
6116	62	20	1	-25
6117	62	21	0	25
6118	62	22	1	-25
6119	62	23	1	25
6120	62	24	1	-25
6121	62	25	1	25
6122	62	26	0	25
6123	62	27	1	-25
6124	62	28	1	25
6125	62	29	0	-25
6126	62	30	0	25
6127	62	31	0	-25
6128	62	32	0	25
6129	62	33	0	-25
6130	62	34	1	-25
6131	62	35	0	25
6132	62	36	0	25
6133	62	37	1	-25
6134	62	38	0	25
6135	62	39	1	-25
6136	62	40	1	-25
6137	62	41	1	25
6138	62	42	0	-25
6139	62	43	0	25

Observation	Subject	Trial	Correct	Pressure
6140	62	44	1	-25
6141	62	45	1	25
6142	62	46	0	25
6143	62	47	0	-25
6144	62	48	1	25
6145	62	49	1	-25
6146	62	50	0	25
6147	62	51	0	0
6148	62	52	1	0
6149	62	53	1	0
6150	62	54	1	0
6151	62	55	0	0
6152	62	56	1	0
6153	62	57	1	0
6154	62	58	1	0
6155	62	59	1	0
6156	62	60	1	0
6157	62	61	1	0
6158	62	62	0	0
6159	62	63	1	0
6160	62	64	1	0
6161	62	65	1	0
6162	62	66	0	0
6163	62	67	1	0
6164	62	68	0	0
6165	62	69	0	0
6166	62	70	1	0
6167	62	71	1	0
6168	62	72	0	0
6169	62	73	1	0
6170	62	74	0	0
6171	62	75	0	0
6172	62	76	1	0
6173	62	77	1	0
6174	62	78	0	0
6175	62	79	1	0
6176	62	80	1	0
6177	62	81	1	0
6178	62	82	1	0
6179	62	83	1	0
6180	62	84	1	0
6181	62	85	1	0
6182	62	86	0	0
6183	62	87	0	0

Observation	Subject	Trial	Correct	Pressure
6184	62	88	0	0
6185	62	89	1	0
6186	62	90	0	0
6187	62	91	0	0
6188	62	92	0	0
6189	62	93	1	0
6190	62	94	0	0
6191	62	95	1	0
6192	62	96	0	0
6193	62	97	0	0
6194	62	98	0	0
6195	62	99	0	0
6196	62	100	0	0
6197	63	1	1	-25
6198	63	2	0	25
6199	63	3	1	-25
6200	63	4	1	-25
6201	63	5	0	25
6202	63	6	1	-25
6203	63	7	1	25
6204	63	8	1	-25
6205	63	9	1	25
6206	63	10	1	25
6207	63	11	1	-25
6208	63	12	0	25
6209	63	13	1	-25
6210	63	14	1	-25
6211	63	15	1	25
6212	63	16	1	-25
6213	63	17	0	25
6214	63	18	1	25
6215	63	19	1	-25
6216	63	20	0	-25
6217	63	21	0	25
6218	63	22	1	-25
6219	63	23	0	25
6220	63	24	1	-25
6221	63	25	1	25
6222	63	26	0	25
6223	63	27	0	-25
6224	63	28	1	25
6225	63	29	1	-25
6226	63	30	0	25
6227	63	31	1	-25

Observation	Subject	Trial	Correct	Pressure
6228	63	32	0	25
6229	63	33	1	-25
6230	63	34	1	-25
6231	63	35	1	25
6232	63	36	0	25
6233	63	37	1	-25
6234	63	38	0	25
6235	63	39	1	-25
6236	63	40	1	-25
6237	63	41	0	25
6238	63	42	1	-25
6239	63	43	1	25
6240	63	44	1	-25
6241	63	45	1	25
6242	63	46	0	25
6243	63	47	1	-25
6244	63	48	1	25
6245	63	49	1	-25
6246	63	50	0	25
6247	63	51	0	0
6248	63	52	1	0
6249	63	53	1	0
6250	63	54	1	0
6251	63	55	0	0
6252	63	56	0	0
6253	63	57	1	0
6254	63	58	1	0
6255	63	59	1	0
6256	63	60	0	0
6257	63	61	1	0
6258	63	62	1	0
6259	63	63	1	0
6260	63	64	1	0
6261	63	65	1	0
6262	63	66	1	0
6263	63	67	1	0
6264	63	68	0	0
6265	63	69	1	0
6266	63	70	1	0
6267	63	71	1	0
6268	63	72	0	0
6269	63	73	1	0
6270	63	74	1	0
6271	63	75	0	0

Observation	Subject	Trial	Correct	Pressure
6272	63	76	1	0
6273	63	77	1	0
6274	63	78	0	0
6275	63	79	0	0
6276	63	80	1	0
6277	63	81	0	0
6278	63	82	1	0
6279	63	83	1	0
6280	63	84	0	0
6281	63	85	0	0
6282	63	86	1	0
6283	63	87	0	0
6284	63	88	0	0
6285	63	89	1	0
6286	63	90	0	0
6287	63	91	0	0
6288	63	92	0	0
6289	63	93	1	0
6290	63	94	0	0
6291	63	95	1	0
6292	63	96	1	0
6293	63	97	0	0
6294	63	98	1	0
6295	63	99	0	0
6296	63	100	0	0
6297	64	1	1	-25
6298	64	2	0	25
6299	64	3	0	-25
6300	64	4	1	-25
6301	64	5	0	25
6302	64	6	1	-25
6303	64	7	1	25
6304	64	8	1	-25
6305	64	9	1	25
6306	64	10	1	25
6307	64	11	1	-25
6308	64	12	1	25
6309	64	13	0	-25
6310	64	14	0	-25
6311	64	15	1	25
6312	64	16	1	-25
6313	64	17	1	25
6314	64	18	0	25
6315	64	19	1	-25

Observation	Subject	Trial	Correct	Pressure
6316	64	20	0	-25
6317	64	21	0	25
6318	64	22	1	-25
6319	64	23	1	25
6320	64	24	1	-25
6321	64	25	0	25
6322	64	26	1	25
6323	64	27	0	-25
6324	64	28	1	25
6325	64	29	1	-25
6326	64	30	0	25
6327	64	31	1	-25
6328	64	32	0	25
6329	64	33	0	-25
6330	64	34	1	-25
6331	64	35	0	25
6332	64	36	0	25
6333	64	37	1	-25
6334	64	38	0	25
6335	64	39	1	-25
6336	64	40	0	-25
6337	64	41	0	25
6338	64	42	0	-25
6339	64	43	1	25
6340	64	44	1	-25
6341	64	45	0	25
6342	64	46	0	25
6343	64	47	1	-25
6344	64	48	1	25
6345	64	49	1	-25
6346	64	50	1	25
6347	64	51	0	0
6348	64	52	1	0
6349	64	53	1	0
6350	64	54	1	0
6351	64	55	1	0
6352	64	56	0	0
6353	64	57	1	0
6354	64	58	1	0
6355	64	59	0	0
6356	64	60	0	0
6357	64	61	1	0
6358	64	62	1	0
6359	64	63	1	0

Observation	Subject	Trial	Correct	Pressure
6360	64	64	0	0
6361	64	65	0	0
6362	64	66	0	0
6363	64	67	1	0
6364	64	68	0	0
6365	64	69	1	0
6366	64	70	0	0
6367	64	71	1	0
6368	64	72	0	0
6369	64	73	1	0
6370	64	74	0	0
6371	64	75	0	0
6372	64	76	1	0
6373	64	77	0	0
6374	64	78	1	0
6375	64	79	0	0
6376	64	80	0	0
6377	64	81	0	0
6378	64	82	1	0
6379	64	83	1	0
6380	64	84	1	0
6381	64	85	1	0
6382	64	86	1	0
6383	64	87	1	0
6384	64	88	0	0
6385	64	89	1	0
6386	64	90	0	0
6387	64	91	0	0
6388	64	92	0	0
6389	64	93	1	0
6390	64	94	0	0
6391	64	95	1	0
6392	64	96	1	0
6393	64	97	0	0
6394	64	98	0	0
6395	64	99	1	0
6396	64	100	1	0
6397	65	1	1	0
6398	65	2	1	0
6399	65	3	1	0
6400	65	4	1	0
6401	65	5	1	0
6402	65	6	1	0
6403	65	7	1	0

Observation	Subject	Trial	Correct	Pressure
6404	65	8	1	0
6405	65	9	1	0
6406	65	10	1	0
6407	65	11	1	0
6408	65	12	0	0
6409	65	13	1	0
6410	65	14	0	0
6411	65	15	1	0
6412	65	16	0	0
6413	65	17	0	0
6414	65	18	1	0
6415	65	19	1	0
6416	65	20	0	0
6417	65	21	1	0
6418	65	22	0	0
6419	65	23	0	0
6420	65	24	0	0
6421	65	25	1	0
6422	65	26	0	0
6423	65	27	0	0
6424	65	28	1	0
6425	65	29	1	0
6426	65	31	1	0
6427	65	32	1	0
6428	65	33	1	0
6429	65	34	1	0
6430	65	35	0	0
6431	65	36	1	0
6432	65	37	1	0
6433	65	38	0	0
6434	65	39	0	0
6435	65	40	1	0
6436	65	41	0	0
6437	65	42	1	0
6438	65	43	1	0
6439	65	44	0	0
6440	65	45	1	0
6441	65	46	0	0
6442	65	47	0	0
6443	65	48	1	0
6444	65	49	1	0
6445	65	50	0	0
6446	65	51	1	-25
6447	65	52	0	25

Observation	Subject	Trial	Correct	Pressure
6448	65	53	1	-25
6449	65	54	1	-25
6450	65	55	1	25
6451	65	56	1	-25
6452	65	57	1	25
6453	65	58	1	-25
6454	65	59	0	25
6455	65	60	0	25
6456	65	61	1	-25
6457	65	62	1	25
6458	65	63	1	-25
6459	65	64	0	-25
6460	65	65	0	25
6461	65	66	0	-25
6462	65	67	0	25
6463	65	68	0	25
6464	65	69	1	-25
6465	65	70	0	-25
6466	65	71	1	25
6467	65	72	0	-25
6468	65	73	1	25
6469	65	74	1	-25
6470	65	75	1	25
6471	65	76	0	25
6472	65	77	1	-25
6473	65	78	1	25
6474	65	79	0	-25
6475	65	80	1	25
6476	65	81	1	-25
6477	65	82	1	25
6478	65	83	1	-25
6479	65	84	1	-25
6480	65	85	0	25
6481	65	86	1	25
6482	65	87	0	-25
6483	65	88	0	25
6484	65	89	1	-25
6485	65	90	0	-25
6486	65	91	1	25
6487	65	92	0	-25
6488	65	93	1	25
6489	65	94	0	-25
6490	65	95	0	25
6491	65	96	1	25

Observation	Subject	Trial	Correct	Pressure
6492	65	97	1	-25
6493	65	98	0	25
6494	65	99	1	-25
6495	65	100	0	25
6496	66	1	1	0
6497	66	2	1	0
6498	66	3	1	0
6499	66	4	1	0
6500	66	5	1	0
6501	66	6	1	0
6502	66	7	1	0
6503	66	8	1	0
6504	66	9	1	0
6505	66	10	1	0
6506	66	11	1	0
6507	66	12	0	0
6508	66	13	0	0
6509	66	14	0	0
6510	66	15	1	0
6511	66	16	1	0
6512	66	17	0	0
6513	66	18	1	0
6514	66	19	0	0
6515	66	20	1	0
6516	66	21	0	0
6517	66	22	0	0
6518	66	23	0	0
6519	66	24	1	0
6520	66	25	1	0
6521	66	26	1	0
6522	66	27	0	0
6523	66	28	1	0
6524	66	29	0	0
6525	66	30	0	0
6526	66	31	1	0
6527	66	32	1	0
6528	66	33	0	0
6529	66	34	1	0
6530	66	35	0	0
6531	66	36	1	0
6532	66	37	1	0
6533	66	38	0	0
6534	66	39	0	0
6535	66	40	0	0

Observation	Subject	Trial	Correct	Pressure
6536	66	41	0	0
6537	66	42	0	0
6538	66	43	0	0
6539	66	44	0	0
6540	66	45	0	0
6541	66	46	0	0
6542	66	47	1	0
6543	66	48	1	0
6544	66	49	1	0
6545	66	50	0	0
6546	66	51	0	-25
6547	66	52	0	25
6548	66	53	0	-25
6549	66	54	1	-25
6550	66	55	0	25
6551	66	56	0	-25
6552	66	57	1	25
6553	66	58	0	-25
6554	66	59	0	25
6555	66	60	1	25
6556	66	61	1	-25
6557	66	62	1	25
6558	66	63	1	-25
6559	66	64	1	-25
6560	66	65	1	25
6561	66	66	0	-25
6562	66	67	0	25
6563	66	68	0	25
6564	66	69	1	-25
6565	66	70	1	-25
6566	66	71	1	25
6567	66	72	0	-25
6568	66	73	1	25
6569	66	74	1	-25
6570	66	75	0	25
6571	66	76	0	25
6572	66	77	1	-25
6573	66	78	1	25
6574	66	79	0	-25
6575	66	80	0	25
6576	66	81	1	-25
6577	66	82	1	25
6578	66	83	1	-25
6579	66	84	1	-25

Observation	Subject	Trial	Correct	Pressure
6580	66	85	0	25
6581	66	86	1	25
6582	66	87	0	-25
6583	66	88	1	25
6584	66	89	0	-25
6585	66	90	1	-25
6586	66	91	0	25
6587	66	92	1	-25
6588	66	93	1	25
6589	66	94	0	-25
6590	66	95	1	25
6591	66	96	1	25
6592	66	97	1	-25
6593	66	98	0	25
6594	66	99	1	-25
6595	66	100	1	25
6596	67	1	0	0
6597	67	2	1	0
6598	67	3	1	0
6599	67	4	1	0
6600	67	5	1	0
6601	67	6	1	0
6602	67	7	1	0
6603	67	8	0	0
6604	67	9	1	0
6605	67	10	1	0
6606	67	11	1	0
6607	67	12	0	0
6608	67	13	1	0
6609	67	14	0	0
6610	67	15	1	0
6611	67	16	0	0
6612	67	17	1	0
6613	67	18	1	0
6614	67	19	1	0
6615	67	20	0	0
6616	67	21	1	0
6617	67	22	1	0
6618	67	23	0	0
6619	67	24	0	0
6620	67	25	1	0
6621	67	26	0	0
6622	67	27	1	0
6623	67	28	1	0

Observation	Subject	Trial	Correct	Pressure
6624	67	29	0	0
6625	67	30	0	0
6626	67	31	1	0
6627	67	32	0	0
6628	67	33	0	0
6629	67	34	1	0
6630	67	35	0	0
6631	67	36	0	0
6632	67	37	1	0
6633	67	38	0	0
6634	67	39	0	0
6635	67	40	1	0
6636	67	41	1	0
6637	67	42	1	0
6638	67	43	1	0
6639	67	44	1	0
6640	67	45	1	0
6641	67	46	0	0
6642	67	47	0	0
6643	67	48	1	0
6644	67	49	1	0
6645	67	50	0	0
6646	67	51	1	-25
6647	67	52	0	25
6648	67	53	1	-25
6649	67	54	1	-25
6650	67	55	0	25
6651	67	56	1	-25
6652	67	57	1	25
6653	67	58	0	-25
6654	67	59	0	25
6655	67	60	0	25
6656	67	61	1	-25
6657	67	62	1	25
6658	67	63	1	-25
6659	67	64	0	-25
6660	67	65	0	25
6661	67	66	1	-25
6662	67	67	0	25
6663	67	68	0	25
6664	67	69	1	-25
6665	67	70	1	-25
6666	67	71	1	25
6667	67	72	1	-25

Observation	Subject	Trial	Correct	Pressure
6668	67	73	1	25
6669	67	74	1	-25
6670	67	75	0	25
6671	67	76	0	25
6672	67	77	1	-25
6673	67	78	1	25
6674	67	79	0	-25
6675	67	80	1	25
6676	67	81	0	-25
6677	67	82	1	25
6678	67	83	1	-25
6679	67	84	1	-25
6680	67	85	0	25
6681	67	86	1	25
6682	67	87	0	-25
6683	67	88	1	25
6684	67	89	1	-25
6685	67	90	0	-25
6686	67	91	1	25
6687	67	92	0	-25
6688	67	93	1	25
6689	67	94	0	-25
6690	67	95	1	25
6691	67	96	1	25
6692	67	97	1	-25
6693	67	98	0	25
6694	67	99	1	-25
6695	67	100	1	25
6696	68	1	0	0
6697	68	2	1	0
6698	68	3	1	0
6699	68	4	1	0
6700	68	5	0	0
6701	68	6	1	0
6702	68	7	1	0
6703	68	8	0	0
6704	68	9	0	0
6705	68	10	1	0
6706	68	11	1	0
6707	68	12	0	0
6708	68	13	0	0
6709	68	14	0	0
6710	68	15	1	0
6711	68	16	1	0

Observation	Subject	Trial	Correct	Pressure
6712	68	17	1	0
6713	68	18	1	0
6714	68	19	0	0
6715	68	20	0	0
6716	68	21	1	0
6717	68	22	1	0
6718	68	23	1	0
6719	68	24	1	0
6720	68	25	0	0
6721	68	26	0	0
6722	68	27	0	0
6723	68	28	1	0
6724	68	29	0	0
6725	68	30	0	0
6726	68	31	1	0
6727	68	32	0	0
6728	68	33	0	0
6729	68	34	1	0
6730	68	35	1	0
6731	68	36	1	0
6732	68	37	1	0
6733	68	38	0	0
6734	68	39	1	0
6735	68	40	1	0
6736	68	41	0	0
6737	68	42	0	0
6738	68	43	1	0
6739	68	44	0	0
6740	68	45	1	0
6741	68	46	0	0
6742	68	47	1	0
6743	68	48	1	0
6744	68	49	1	0
6745	68	50	0	0
6746	68	51	1	-25
6747	68	52	0	25
6748	68	53	0	-25
6749	68	54	1	-25
6750	68	55	0	25
6751	68	56	0	-25
6752	68	57	1	25
6753	68	58	0	-25
6754	68	59	1	25
6755	68	60	0	25

Observation	Subject	Trial	Correct	Pressure
6756	68	61	1	-25
6757	68	62	1	25
6758	68	63	1	-25
6759	68	64	0	-25
6760	68	65	1	25
6761	68	66	0	-25
6762	68	67	1	25
6763	68	68	0	25
6764	68	69	1	-25
6765	68	70	0	-25
6766	68	71	1	25
6767	68	72	0	-25
6768	68	73	1	25
6769	68	74	1	-25
6770	68	75	0	25
6771	68	76	1	25
6772	68	77	1	-25
6773	68	78	0	25
6774	68	79	0	-25
6775	68	80	1	25
6776	68	81	0	-25
6777	68	82	1	25
6778	68	83	1	-25
6779	68	84	1	-25
6780	68	85	0	25
6781	68	86	1	25
6782	68	87	0	-25
6783	68	88	1	25
6784	68	89	0	-25
6785	68	90	1	-25
6786	68	91	0	25
6787	68	92	0	-25
6788	68	93	1	25
6789	68	94	0	-25
6790	68	95	1	25
6791	68	96	1	25
6792	68	97	1	-25
6793	68	98	0	25
6794	68	99	1	-25
6795	68	100	0	25
6796	69	1	1	-25
6797	69	2	0	25
6798	69	3	0	-25
6799	69	4	1	-25

Observation	Subject	Trial	Correct	Pressure
6800	69	5	0	25
6801	69	6	1	-25
6802	69	7	0	25
6803	69	8	1	-25
6804	69	9	1	25
6805	69	10	1	25
6806	69	11	1	-25
6807	69	12	1	25
6808	69	13	0	-25
6809	69	14	0	-25
6810	69	15	1	25
6811	69	16	1	-25
6812	69	17	1	25
6813	69	18	1	25
6814	69	19	1	-25
6815	69	20	0	-25
6816	69	21	1	25
6817	69	22	0	-25
6818	69	23	1	25
6819	69	24	1	-25
6820	69	25	1	25
6821	69	27	0	-25
6822	69	28	1	25
6823	69	29	0	-25
6824	69	30	0	25
6825	69	31	1	-25
6826	69	32	1	25
6827	69	33	1	-25
6828	69	34	1	-25
6829	69	35	1	25
6830	69	36	1	25
6831	69	37	1	-25
6832	69	38	0	25
6833	69	39	1	-25
6834	69	40	1	-25
6835	69	41	1	25
6836	69	42	0	-25
6837	69	43	1	25
6838	69	44	0	-25
6839	69	45	1	25
6840	69	46	1	25
6841	69	47	1	-25
6842	69	48	1	25
6843	69	49	1	-25

Observation	Subject	Trial	Correct	Pressure
6844	69	50	0	25
6845	69	51	0	0
6846	69	52	0	0
6847	69	53	1	0
6848	69	54	1	0
6849	69	55	0	0
6850	69	56	1	0
6851	69	57	1	0
6852	69	58	0	0
6853	69	59	0	0
6854	69	60	0	0
6855	69	61	1	0
6856	69	62	1	0
6857	69	63	1	0
6858	69	64	0	0
6859	69	65	0	0
6860	69	66	0	0
6861	69	67	1	0
6862	69	68	1	0
6863	69	69	1	0
6864	69	70	1	0
6865	69	71	1	0
6866	69	72	1	0
6867	69	73	0	0
6868	69	74	1	0
6869	69	75	0	0
6870	69	76	0	0
6871	69	77	1	0
6872	69	78	1	0
6873	69	79	0	0
6874	69	80	1	0
6875	69	81	1	0
6876	69	82	1	0
6877	69	83	1	0
6878	69	84	1	0
6879	69	85	0	0
6880	69	86	1	0
6881	69	87	0	0
6882	69	88	0	0
6883	69	89	1	0
6884	69	90	1	0
6885	69	91	0	0
6886	69	92	0	0
6887	69	93	1	0

Observation	Subject	Trial	Correct	Pressure
6888	69	94	1	0
6889	69	95	1	0
6890	69	96	1	0
6891	69	97	1	0
6892	69	98	0	0
6893	69	99	1	0
6894	69	100	0	0
6895	70	1	1	-25
6896	70	2	0	25
6897	70	3	1	-25
6898	70	4	1	-25
6899	70	5	1	25
6900	70	6	0	-25
6901	70	7	1	25
6902	70	8	1	-25
6903	70	9	1	25
6904	70	10	0	25
6905	70	11	1	-25
6906	70	12	0	25
6907	70	13	1	-25
6908	70	14	0	-25
6909	70	15	1	25
6910	70	16	1	-25
6911	70	17	0	25
6912	70	18	1	25
6913	70	19	1	-25
6914	70	20	0	-25
6915	70	21	1	25
6916	70	22	0	-25
6917	70	23	1	25
6918	70	24	1	-25
6919	70	25	0	25
6920	70	26	0	25
6921	70	27	0	-25
6922	70	28	1	25
6923	70	29	0	-25
6924	70	30	1	25
6925	70	31	1	-25
6926	70	32	0	25
6927	70	33	0	-25
6928	70	34	1	-25
6929	70	35	1	25
6930	70	36	0	25
6931	70	37	1	-25

Observation	Subject	Trial	Correct	Pressure
6932	70	38	0	25
6933	70	39	1	-25
6934	70	40	1	-25
6935	70	41	0	25
6936	70	42	1	-25
6937	70	43	1	25
6938	70	44	0	-25
6939	70	45	1	25
6940	70	46	0	25
6941	70	47	1	-25
6942	70	48	1	25
6943	70	49	1	-25
6944	70	50	1	25
6945	70	51	1	0
6946	70	52	0	0
6947	70	53	1	0
6948	70	54	1	0
6949	70	55	1	0
6950	70	56	0	0
6951	70	57	1	0
6952	70	58	1	0
6953	70	59	0	0
6954	70	60	1	0
6955	70	61	1	0
6956	70	62	1	0
6957	70	63	1	0
6958	70	64	0	0
6959	70	65	1	0
6960	70	66	0	0
6961	70	67	0	0
6962	70	68	1	0
6963	70	69	1	0
6964	70	70	1	0
6965	70	71	1	0
6966	70	72	0	0
6967	70	73	1	0
6968	70	74	1	0
6969	70	75	0	0
6970	70	76	1	0
6971	70	77	0	0
6972	70	78	1	0
6973	70	79	0	0
6974	70	80	1	0
6975	70	81	1	0

Observation	Subject	Trial	Correct	Pressure
6976	70	82	1	0
6977	70	83	1	0
6978	70	84	1	0
6979	70	85	0	0
6980	70	86	1	0
6981	70	87	0	0
6982	70	88	1	0
6983	70	89	0	0
6984	70	90	0	0
6985	70	91	0	0
6986	70	92	1	0
6987	70	93	1	0
6988	70	94	0	0
6989	70	95	1	0
6990	70	96	0	0
6991	70	97	0	0
6992	70	98	1	0
6993	70	99	1	0
6994	70	100	0	0
6995	71	1	1	-25
6996	71	2	0	25
6997	71	3	1	-25
6998	71	4	1	-25
6999	71	5	0	25
7000	71	6	1	-25
7001	71	7	1	25
7002	71	8	1	-25
7003	71	9	1	25
7004	71	10	0	25
7005	71	11	1	-25
7006	71	12	0	25
7007	71	13	1	-25
7008	71	14	1	-25
7009	71	15	1	25
7010	71	16	0	-25
7011	71	17	1	25
7012	71	18	1	25
7013	71	19	1	-25
7014	71	20	0	-25
7015	71	21	0	25
7016	71	22	1	-25
7017	71	23	1	25
7018	71	24	1	-25
7019	71	25	0	25

Observation	Subject	Trial	Correct	Pressure
7020	71	26	0	25
7021	71	27	0	-25
7022	71	28	1	25
7023	71	29	0	-25
7024	71	30	0	25
7025	71	31	1	-25
7026	71	32	0	25
7027	71	33	0	-25
7028	71	34	1	-25
7029	71	35	1	25
7030	71	36	1	25
7031	71	37	1	-25
7032	71	38	0	25
7033	71	39	1	-25
7034	71	40	1	-25
7035	71	41	0	25
7036	71	42	1	-25
7037	71	43	1	25
7038	71	44	0	-25
7039	71	45	0	25
7040	71	46	1	25
7041	71	47	0	-25
7042	71	48	1	25
7043	71	49	1	-25
7044	71	50	0	25
7045	71	51	1	0
7046	71	52	0	0
7047	71	53	1	0
7048	71	54	1	0
7049	71	55	0	0
7050	71	56	0	0
7051	71	57	1	0
7052	71	58	1	0
7053	71	59	0	0
7054	71	60	0	0
7055	71	61	1	0
7056	71	62	1	0
7057	71	63	1	0
7058	71	64	1	0
7059	71	65	1	0
7060	71	66	0	0
7061	71	67	1	0
7062	71	68	0	0
7063	71	69	1	0

Observation	Subject	Trial	Correct	Pressure
7064	71	70	1	0
7065	71	71	1	0
7066	71	72	0	0
7067	71	73	1	0
7068	71	74	0	0
7069	71	75	0	0
7070	71	76	0	0
7071	71	77	1	0
7072	71	78	1	0
7073	71	79	0	0
7074	71	80	1	0
7075	71	81	0	0
7076	71	82	1	0
7077	71	83	1	0
7078	71	84	0	0
7079	71	85	1	0
7080	71	86	1	0
7081	71	87	0	0
7082	71	88	1	0
7083	71	89	1	0
7084	71	90	0	0
7085	71	91	0	0
7086	71	92	1	0
7087	71	93	1	0
7088	71	94	0	0
7089	71	95	1	0
7090	71	96	1	0
7091	71	97	1	0
7092	71	98	1	0
7093	71	99	0	0
7094	71	100	0	0
7095	72	1	1	-25
7096	72	2	0	25
7097	72	3	1	-25
7098	72	4	1	-25
7099	72	5	1	25
7100	72	6	1	-25
7101	72	7	1	25
7102	72	8	1	-25
7103	72	9	1	25
7104	72	10	1	25
7105	72	11	1	-25
7106	72	12	0	25
7107	72	13	0	-25

Observation	Subject	Trial	Correct	Pressure
7108	72	14	0	-25
7109	72	15	1	25
7110	72	16	1	-25
7111	72	17	1	25
7112	72	18	1	25
7113	72	19	0	-25
7114	72	20	1	-25
7115	72	21	0	25
7116	72	22	1	-25
7117	72	23	0	25
7118	72	24	1	-25
7119	72	25	0	25
7120	72	26	1	25
7121	72	27	0	-25
7122	72	28	1	25
7123	72	29	0	-25
7124	72	30	0	25
7125	72	31	1	-25
7126	72	32	0	25
7127	72	33	0	-25
7128	72	34	1	-25
7129	72	35	0	25
7130	72	36	0	25
7131	72	37	1	-25
7132	72	38	1	25
7133	72	39	1	-25
7134	72	40	1	-25
7135	72	41	1	25
7136	72	42	0	-25
7137	72	43	1	25
7138	72	44	1	-25
7139	72	45	1	25
7140	72	46	0	25
7141	72	47	0	-25
7142	72	48	1	25
7143	72	49	1	-25
7144	72	50	0	25
7145	72	51	0	0
7146	72	52	1	0
7147	72	53	1	0
7148	72	54	1	0
7149	72	55	1	0
7150	72	56	0	0
7151	72	57	1	0

Observation	Subject	Trial	Correct	Pressure
7152	72	58	1	0
7153	72	59	1	0
7154	72	60	1	0
7155	72	61	1	0
7156	72	62	1	0
7157	72	63	1	0
7158	72	64	0	0
7159	72	65	1	0
7160	72	66	0	0
7161	72	67	1	0
7162	72	68	0	0
7163	72	69	1	0
7164	72	70	0	0
7165	72	71	1	0
7166	72	72	1	0
7167	72	73	0	0
7168	72	74	0	0
7169	72	75	1	0
7170	72	76	0	0
7171	72	77	1	0
7172	72	78	0	0
7173	72	79	0	0
7174	72	80	1	0
7175	72	81	0	0
7176	72	82	1	0
7177	72	83	1	0
7178	72	84	0	0
7179	72	85	0	0
7180	72	86	1	0
7181	72	87	0	0
7182	72	88	0	0
7183	72	89	0	0
7184	72	90	0	0
7185	72	91	1	0
7186	72	92	0	0
7187	72	93	1	0
7188	72	94	0	0
7189	72	95	1	0
7190	72	96	1	0
7191	72	97	0	0
7192	72	98	0	0
7193	72	99	0	0
7194	72	100	1	0
